# Novel clades of soil biphenyl degraders revealed by integrating isotope probing, multi-omics, and single-cell analyses

**DOI:** 10.1038/s41396-021-01022-9

**Published:** 2021-06-11

**Authors:** Song-Can Chen, Rohit Budhraja, Lorenz Adrian, Federica Calabrese, Hryhoriy Stryhanyuk, Niculina Musat, Hans-Hermann Richnow, Gui-Lan Duan, Yong-Guan Zhu, Florin Musat

**Affiliations:** 1 Department of Isotope Biogeochemistry, Helmholtz Centre for Environmental Research–UFZ, 04318 Leipzig, Germany; 2 Department Environmental Biotechnology, Helmholtz Centre for Environmental Research–UFZ, Leipzig, Germany; 3 Chair of Geobiotechnology, Technische Universität Berlin, 13355 Berlin, Germany; 4 State Key Lab of Urban and Regional Ecology, Research Center for Eco-Environmental Sciences, Chinese Academy of Sciences, 100085 Beijing, China; 5 Key Lab of Urban Environment and Health, Institute of Urban Environment, Chinese Academy of Sciences, 361021 Xiamen, China

**Keywords:** Microbial ecology, Biogeochemistry, Biogeochemistry, Soil microbiology, Biogeochemistry

## Abstract

Most microorganisms in the biosphere remain uncultured and poorly characterized. Although the surge in genome sequences has enabled insights into the genetic and metabolic properties of uncultured microorganisms, their physiology and ecological roles cannot be determined without direct probing of their activities in natural habitats. Here we employed an experimental framework coupling genome reconstruction and activity assays to characterize the largely uncultured microorganisms responsible for aerobic biodegradation of biphenyl as a proxy for a large class of environmental pollutants, polychlorinated biphenyls. We used ^13^C-labeled biphenyl in contaminated soils and traced the flow of pollutant-derived carbon into active cells using single-cell analyses and protein–stable isotope probing. The detection of ^13^C-enriched proteins linked biphenyl biodegradation to the uncultured *Alphaproteobacteria* clade UBA11222, which we found to host a distinctive biphenyl dioxygenase gene widely retrieved from contaminated environments. The same approach indicated the capacity of *Azoarcus* species to oxidize biphenyl and suggested similar metabolic abilities for species of *Rugosibacter*. Biphenyl oxidation would thus represent formerly unrecognized ecological functions of both genera. The quantitative role of these microorganisms in pollutant degradation was resolved using single-cell-based uptake measurements. Our strategy advances our understanding of microbially mediated biodegradation processes and has general application potential for elucidating the ecological roles of uncultured microorganisms in their natural habitats.

## Introduction

One of the grand challenges of microbial ecology and ecosystem science is to link microbial community structure to microbial functional traits underpinning key ecological processes [1, 2]. To date, the physiological and ecological functions of most microorganisms in natural habitats remain poorly characterized particularly due to their recalcitrance to cultivation [3, 4]. To some extent, the need to cultivate individual taxa has been overcome by recent advances in environmental metagenomic sequencing and computational approaches. This enabled community-level genome reconstruction from a wide range of ecosystems, shedding new insights into phylogenetic identity and metabolic potential of numerous uncultured lineages [5–9]. Consequently, the physiology and ecological roles of uncultured bacteria and archaea have been lately inferred mostly from reconstructed genomes. However, these genome-driven discoveries relied strongly on the predictive understanding of genes and metabolic pathways, which do not always reflect actual functions carried out by microorganisms in their natural habitats. Thus, it became apparent that experimental evidence to support genome-based predictions by direct detection and quantification of function is needed to properly understand the role of uncultured microorganisms in ecologically significant biogeochemical processes [2, 4, 10, 11].

A prominent, microbially mediated environmental process is the biodegradation of widespread, persistent organic pollutants like polychlorinated biphenyls (PCBs), which have broad consequences for human health and ecosystem functioning [12, 13]. The identity of microorganisms involved in such processes in various environments has been extensively investigated by both culture-dependent and culture-independent methods. The formerly provided insights into the biochemical basis of pollutant activation and downstream transformation in pure cultures [14–17]. The latter targeted active pollutant-metabolizing microorganisms within natural microbial communities, such as those inhabiting PCB-contaminated soils and sediments, through substrate-specific labeling of their DNA by stable isotope probing (SIP). Sequence analysis of marker genes (i.e., 16S rRNA gene) retrieved from labeled fractions of community DNA revealed the phylogenetic identity of metabolically active populations [18–23]. These SIP-based gene surveys indicated that pollutant removal in contaminated environments was mostly achieved by uncultured microorganisms; only a small fraction of these are represented by closely related isolates or genomes [24–26]. Thus, there is remarkably little reliable information about the full genetic potential and taxon-specific metabolism of the ecologically relevant degraders. Furthermore, DNA-based SIP provided only a qualitative link between phylogenetic identity and biodegradation activity, whereas direct quantification of metabolic rate and cellular abundance has never been obtained for pollutant degraders in real-world habitats like soils.

Here we selected biphenyl as a model compound to document metabolic activities and genomic makeup of biodegradative microbial populations of PCB-impacted soils. Biphenyl has often been used as a proxy to investigate the biodegradation of PCBs since many PCB congeners can be co-metabolized by biphenyl-oxidizing microorganisms via the same oxidation pathways [27, 28]. The capacity of the soil microbial communities to oxidize biphenyl was initially assessed using cultivation approaches. We then coupled stable isotope probing with nanoscale secondary ion mass spectrometry (nanoSIMS) to image and quantify biphenyl assimilation by individual microbial cells. The phylogenetic identity of biphenyl oxidizers was resolved using metaproteomics-SIP based on metagenomic databases constructed for each soil type investigated. Moreover, for the identified microorganisms, we used the metagenome-assembled genomes (MAGs) to reconstruct the complete metabolic pathways involved in biphenyl oxidization.

## Materials and methods

### Chemicals

The chemicals used include ^13^C-biphenyl (99 atom% ^13^C; Cambridge Isotope Laboratories, Inc., USA), unlabeled biphenyl (Sigma-Aldrich), nuclease-free water (ThermoFisher Scientific), dichloromethane (Sigma-Aldrich), sodium cacodylate buffer (Electron Microscopy Sciences, USA), and Nycodenz (Progen).

### Soil samples

The four soil samples investigated in this study were collected from Taizhou, Zhejiang Province, China, and were denoted as soil A (river sediment; 28°32’19”N, 121°22’41”E), soil B (paddy soil; 28°32’28”N, 121°22’15”E), soil C (river sediment; 28°37’13”N, 121°24’54”E), and soil D (upland soil; 28°30’32”N, 121°21’49”E), respectively. The sampling sites were historically contaminated with PCBs at concentrations of 0.03 − 5 mg kg^−1^ (Supplementary Table 1). The samples were stored at 4 °C until analysis or use for incubation experiments. A diagram summarizing our main experimental workflow is presented in Supplementary Fig. 1.

### Establishing biphenyl-degrading enrichment cultures

Incubations with biphenyl were set up in 150 ml conical flasks provided with 30 ml aerobic, mineral freshwater medium [29, 30], and 5 g of soil as inoculum. Crystals of biphenyl (approx. 20 mg per flask) were added as the sole carbon and energy source. The flasks were sealed with cotton stoppers to permit the free passage of oxygen. Incubation was done at 28 °C in the dark with gentle horizontal shaking (120 rpm). Controls without the addition of biphenyl were prepared similarly and incubated under the same conditions. Biphenyl-dependent growth was monitored by observing changes of turbidity and by microscopic examinations. Sediment-free enrichment cultures were obtained after five subsequent transfers into fresh culture media with 10% inoculum each. Enrichment cultures were further maintained by inoculating 10% volume of grown cultures to fresh-water medium every three days. Growth experiments with the enrichment cultures were prepared under the same conditions as described above. Growth was monitored by measuring the OD at *λ* = 600 nm every four hours by subsampling 0.5 ml of culture. Inoculated medium without biphenyl was used as a control.

### DNA extraction and community sequencing

DNA was extracted from 0.5 g soil as described previously [31]. The soil samples were suspended in 1.35 ml extraction buffer (100 mM Tris-HCl, 100 mM sodium EDTA, 100 mM sodium phosphate, 1.5 M NaCl, 1% CTAB, pH 8.0) and lysed via three cycles of freezing and thawing at −196 °C and 37 °C, respectively. The resulting lysates were further incubated with 10 mg ml^−1^ proteinase K at 37 °C for 30 min and with 20% SDS at 65 °C for 1 h, followed by 10 min centrifugation at 12 °C, 15,000 rpm (21,130×*g*). The resulting supernatant was extracted with an equal volume of chloroform:isoamyl alcohol (24:1, v/v). Nucleic acids were precipitated with isopropanol (0.6 volume, 1 h at room temperature). The yielded DNA was dissolved in 50 μl nuclease-free water and stored at −20 °C before use. For DNA extraction from biphenyl-degrading enrichments, the same procedure was applied to cells harvested from 20 ml grown cultures. The V3-V5 region of bacterial 16S rRNA genes was amplified using primers 341F (5’-CCTACGGGNGGCWGCAG-3’) and 785 R (5’-GACTACHVGGGTATCTAATCC-3’) and sequenced on MiSeq (Illumina) V3 platform (2 × 300 cycles). After removal of adapters and primers, the paired-end reads were joined using BBMap v38.51 (https://sourceforge.net/projects/bbmap/) and processed using the SilvaNGS pipeline [32, 33]. Operational taxonomic units (OTUs) were clustered at 98% sequence identity level and classified taxonomically using the Silva v.132 databases. The OTU table was downloaded from SilvaNGS and rarefied to minimum sequencing depth across samples (36,000 for soils; 10,000 for enrichments). Alpha diversity indices, including the Shannon, Chao1, Simpson, and Simpson’s evenness measure E, were calculated using the Vegan R package (Supplementary Table 2). Beta diversity was assessed using the *capscale* function in the Phyloseq R package based on Bray-Curtis dissimilarity (Supplementary Fig. 2).

### Stable isotope probing in slurry microcosms

To determine the biphenyl-degrading capacity of the original soil samples, slurry microcosms were established in 20-ml sealed serum vials. To enhance biphenyl bioavailability, unlabeled or ^13^C-labeled biphenyl was dissolved in dichloromethane (DCM) at a concentration of 20 mg/ml. A volume of 0.1 ml of this solution was added per vial (2 mg net biphenyl per vial); the DCM was thereafter completely evaporated, leaving biphenyl crystals homogeneously distributed on the inner wall of vials. Soil slurries were prepared for each soil type by mixing soil samples with freshwater medium to reach ~75% (v/v) pore-water content. Vials were thereafter supplied with 3 ml slurry, sealed with rubber stoppers, and incubated in the dark at 28 °C with continuous shaking (120 rpm). For each soil type, a total of 14 replicate microcosms were prepared, of which 7 contained unlabeled biphenyl and 7 contained ^13^C-biphenyl. Two replicates were sacrificed for each soil type and substrate combination, at seven time points (0, 2, 4, 8, 24, 48, and 96 h of incubation). The headspace of the microcosm was collected to determine the carbon isotope composition of CO_2_ [34]. The slurries were either frozen (−20 °C) for molecular biological analysis or fixed for nanoSIMS analysis. For sample fixation, the sediments were incubated with 3% paraformaldehyde (final concentration) for 1 h at room temperature, washed two times with 1× PBS, and stored in 1× PBS:ethanol (1:1, v/v) at −20 °C.

### Soil cell extraction and nanoSIMS analysis

To detach cells from soil particles for subsequent nanoSIMS analyses, we used established protocols based on alternating mild chemicals (using tensioactive compounds) and mechanical treatments [35]. In brief, 200 μl of PFA-fixed sediment was mixed with 700 μl TE buffer (10 mM Tris-HCl, 5 mM EDTA, pH 9.0) and 100 μl pyrophosphate (100 mM), followed by incubation at 55 °C for 5 min (water bath of a histological microwave oven operated at 200 W; Microwave Research and Application Inc., Laurel, MD, USA). The samples were then amended with 1 μl Tween 80 and mixed for 15 min at room temperature on a benchtop vortex (level 3, 1060 rpm, Grant-bio). Cells were thereafter separated from soil particles using established Nycodenz density centrifugation protocols [35]. Each sample was transferred to a 50 ml Falcon tube containing 22.5 ml 1× PBS and 2.5 ml pyrophosphate. A volume of 2 ml Nycodenz solution (1.426 g/ml, 80% w/v) was slowly added to the bottom of the Falcon tube using 10 ml pipette tips to avoid mixing of Nycodenz and slurries. High-speed centrifugation (1.5 h, 16,000×*g*, 4 °C; ROTINA 380R, Hettich) was applied to separate cells from soil particles. Cell-containing water phase (approx. 25 ml) on the top of the Nycodenz layer was carefully collected in clean 50 ml Falcon tubes.

For nanoSIMS analyses, 10 ml of supernatant recovered from the Nycodenz extraction procedure was filtered on gold-palladium-coated polycarbonate filters (0.22 μm pore size). The samples were rinsed twice with sodium cacodylate buffer (0.2 M, pH 7.4; Electron Microscopy Sciences), dehydrated in an ethanol series prepared in the same buffer (30, 50, 70, 80, 90, 96, and 100%), and dried for 20 exchange cycles using critical point drying machine (Critical Point Dryer, LEICA EM CPD300). Filter pieces of 10 mm in diameter were cut using a punching tool and placed on the sample holder of a nanoSIMS-50L instrument (CAMECA, AMETEK). NanoSIMS analyses were performed in negative extraction mode using Cs^+^ as the primary ion source. Prior to measurements, 100 × 100 μm^2^ areas were pre-implanted with 16 keV cesium (Cs^+^) primary ion beam at 200 pA for 10 min. Fields of view of 25 × 25 μm^2^ were analyzed with a 3 pA primary ion beam, scanning at 512 × 512-pixel resolution with 2 ms per pixel of dwell time. Secondary ion species (^12^CH^−^, ^16^O^−^, ^12^C^14^N^−^, ^13^C^14^N^−^, ^31^P^−^, ^32^S^−^, and ^31^P^16^O_2_^−^) were collected in parallel. A mass resolving power (M/ΔM) between 8000 and 12,000 was achieved as previously described, with 40 μm exit slits. Look@NanoSIMS software (LANS) was used to process the acquired secondary ion images [36]. More than 25 scans of each field of view were accumulated after lateral drift corrections. Region of interest (RoI) was manually defined for each individual cell along its inner margin based on ^12^C^14^N^−^ ion images. The raw ion counts of ^13^C^14^N^−^ and ^12^C^14^N^−^ were used to calculate the fraction of C assimilated by a single cell (K_A_). Assimilation rates of carbon (F_c_) were derived for individual cells as described previously [37]. The total number of analyzed cells and those showing ^13^C-enrichment in each soil sample are summarized in Supplementary Table 3.

### Metagenomic sequencing and data analysis

DNA extracted from unlabeled soil microcosms after 96 h incubation was sequenced on a MiSeq (Illumina) V3 platform (2×300 cycles), resulting in >2 million paired-end reads for each soil sample (*n* = 4). The raw reads were quality filtered using Trimmomatic v.0.33 [38] (SLIDINGWINDOW:4:15; MINLEN 36) and assembled into contigs using Spades v3.14.0 [39] with the following the parameters (-meta; -k 21,33,55,77,99,127). Short contigs ≤500 bp were removed. Metagenomic binning was performed using MetaBAT v0.32.4 [40] with -minContig option of 1500. The generated MAGs were further refined using RefineM v0.0.25 [7] based on genomic properties (i.e., tetranucleotide frequency and read coverage) and taxonomic classification. MAGs of interest were further refined by reads mapping, reassembly, and binning. Reads were mapped to the selected MAGs at a minimum alignment identity of 90% using BBMap v.38.51 (minid option). Reassembly and binning followed the same pipeline described above. The quality (completeness and contamination) of each MAG was assessed using CheckM v1.1.2 [41] with the lineage-specific workflow. Taxonomy assignments of MAGs were conducted using GTDB-Tk [42]. The genome tree of bacterial MAGs was inferred from the concatenation of 120 proteins using RAxML v8.2.11 [43] with 100 bootstrap replicates (-m PROTCAMMALG -f a -N 100). For MAGs, protein-coding genes were called using Prodigal [44] (-p single option) and the translated protein sequences were annotated with KEGG [45], Pfam [46], and EggNOG [47] databases. To search for aerobic biphenyl-oxidation pathways in genomes, reference protein sequences from the *bph* operon of various known biphenyl degraders, including Gram-positive and Gram-negative bacteria (Supplementary Table 4), were queried against MAGs using Blastp [48] (E-value, 1e-20; minimum sequence identity, 30%). The resulting blast hits were manually curated based on sequence length, domain composition, and conserved functional sites. To reconstruct the phylogenetic tree of BphA, protein sequences were aligned using Muscle v3.8.1551 [49], followed by trimming of ambiguous sites in the alignment using trimAl v1.4 [50] with *-automated1* option. Maximum likelihood trees were calculated using RAxML with the PROTGAMMALG evolutionary model.

### Metaproteomics and protein stable isotope probing

In the protein-SIP experiment, soils incubated with unlabeled and labeled biphenyl (20 atom% ^13^C) were processed in parallel. Extraction and quantification of proteins from soil microcosms (3 ml) were carried out using NoviPure Soil Protein Kit (QIAGEN) and BCA Protein Assay Kit (QIAGEN), respectively. The extracted proteins were separated by sodium dodecyl sulfate-polyacrylamide gel electrophoresis (SDS-PAGE). For each sample, the corresponding SDS-PAGE gel lane was cut into two pieces. Protein in the gel pieces was reduced with dithionite, carbamidomethylated with iodoacetamide, and digested with trypsin as described by Budhraja et al (2021). Peptides were purified using ZipTip C18-columns (Millipore) and analyzed by an Orbitrap Fusion Tribrid mass spectrometer (Thermo Scientific) equipped with a nanoLC system (Dionex Ultimate 3000RSLC; Thermo Scientific) [8, 51].

The obtained MS/MS spectra were searched against the specific metagenome database (bulk assembly) by XTandem [52] in the OpenMS pipeline [53]. Precursor and fragment ion mass tolerance were set to 5 ppm and 0.02 Da, respectively. Two tryptic miss cleavages were allowed; carbamidomethylation of cysteines was treated as fixed modification and oxidation of methionine as a dynamic modification. Proteins were considered to be identified when at least two unique peptides were recovered with medium confidence (False discovery rate, FDR < 0.05).

Incorporation of ^13^C was determined for peptides at a high confidence level (FDR < 0.01) using MetaProSIP [54] with a mass window of 10 ppm. Centroid MS1 data, the metagenome database, and feature data annotated with identification (generated by IDMapper in OpenMS) were fed as input for MetaProSIP. A minimum correlation of 0.8 was set as the threshold for reporting ^13^C-peptides to reduce false positives. Relative isotope abundance (RIA) and labeling ratio (LR) of peptides were retrieved for labeled peptides from the output of MetaProSIP. The isotope pattern of the labeled peptides was further evaluated by comparing the observed mass spectra with its theoretical distributions using Pearson’s chi-squared test (*χ*^2^) in R software.

## Results

### Soil microbial diversity and biphenyl biodegradation potential

**Fig. 1:**
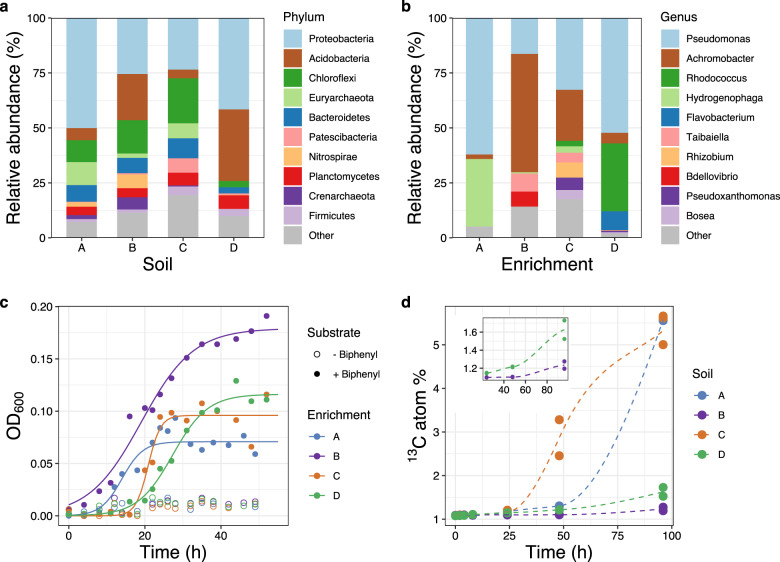
Biphenyl biodegradation potential in the four contaminated soils. **a**–**b** Relative abundance of major microbial lineages in each soil (**a**) and in the corresponding biphenyl-degrading enrichment cultures (**b**). **c** Growth curves of the biphenyl-degrading enrichment cultures when amended with biphenyl (filled circles), vs. control incubations (without biphenyl, open circles). Cultures A and C formed large flocs after 24 h of incubation, which led to high OD scattering. **d** Evolution of ^13^CO_2_ in the headspace of soil microcosms incubated with ^13^C-biphenyl. The last three times points of soil B and D were replotted as an inset to show the mineralization of ^13^C-biphenyl. Duplicate assays were done for each soil type. For soil A, only one of the replicates was active.

Soil samples were collected at four different sites with a long history of contamination with aromatic compounds. Polychlorinated biphenyls (PCBs) were among the most prominent contaminants (Supplementary Table 1). Since microorganisms often use similar biochemical pathways to oxidize both biphenyl and PCBs [16], we hypothesized that the sampled soils contained biphenyl-oxidizing microorganisms. Amplicon sequencing of 16S rRNA genes indicated that all four soils harbored highly diverse microbial communities. A total of 18,188, 29,517, 49,167, and 35,121 operational taxonomic units (OTUs) were observed for soils A, B, C, and D, respectively. Among these, OTUs affiliated with *Proteobacteria* accounted for more than 20% of all sequences in each soil type. The remaining bacterial sequences were assigned to *Acidobacteria* (4–33%), *Chloroflexi* (3–20%), *Bacteroidetes* (3–9%), and *Planctomycetes* (4–6%) (Fig. 1a). Archaea (mostly *Euryarchaeota* and *Crenarchaeota*) contributed to 8–12% of total sequences in soils A, B, C, and less than 0.5% in soil D. Overall, the dominant phyla detected here matched the microbial diversity typically associated with PCB-contaminated soils [55].

Cultivation experiments showed that all soils had the potential to oxidize biphenyl, with biphenyl-dependent microbial growth observed within 5 to 10 days of incubation. Sediment-free biphenyl-oxidizing enrichment cultures were obtained from each soil type. These cultures formed typical yellow meta-cleavage intermediates during growth (not shown) [56]. The approximate doubling time calculated from the OD-based growth curves ranged from 1.6 h to 5.7 h. Taxonomic profiling based on 16S rRNA genes revealed that the enrichments were dominated by *Pseudomonas*, *Achromobacter*, *Rhodococcus*, *Hydrogenophaga*, and *Flavobacterium* (Fig. 1b). Members of these genera have been frequently shown to degrade biphenyl [12], suggesting a similar function in the obtained cultures.

Short-term labeling experiments with ^13^C-biphenyl demonstrated that biphenyl-oxidizing microorganisms were present and active in all original soils. The formation of ^13^CO_2_ was detected in microcosms of all soil types, albeit at different extent, indicating different biphenyl mineralization rates. The fastest response was measured for soil C, where ^13^CO_2_ was detected within 24 h and continued to increase in concentration throughout the experiment, reaching 5.2 atom% labelings within 4 days of incubation (Fig. 1d). In contrast, longer lag phases and lower abundances of ^13^CO_2_ were observed for soils A, B, and D.

### Visualization of soil biphenyl-degraders at a single-cell level

**Fig. 2:**
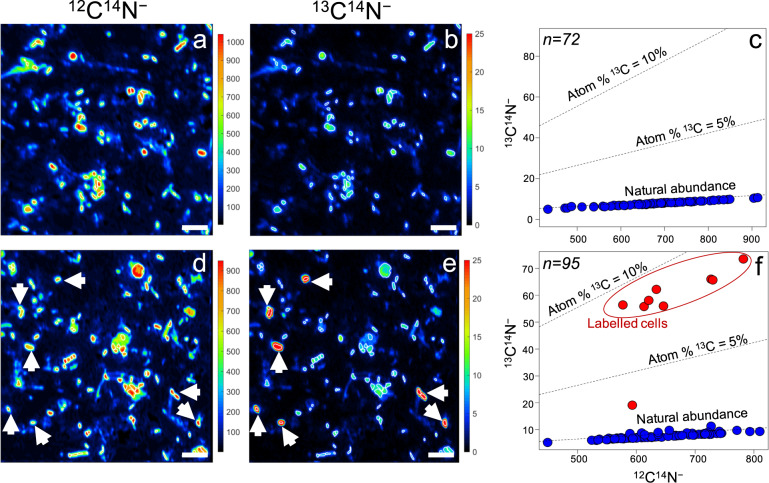
NanoSIMS analysis of cells extracted from soil C after incubation with ^13^C-labeled biphenyl for 0 (a–c) and 96 h (d–f). Secondary ion images of ^12^C^14^N^−^ (**a**, **d**) and ^13^C^14^N^−^ (**b**, **e**) were used to identify individual cells and to quantify ^13^C abundance. Arrowhead in ^12^C^14^N^−^ ion image (**d**) point to the cells enriched in ^13^C in panel **e**. Raw ion counts of ^12^C^14^N^−^ and ^13^C^14^N^−^ for individual cells were shown as scatter plots (**c**, **f**). Cells having ^13^C abundance over 2% are presented as red symbols (**f**), whereas other cells are presented as blue symbols (**c**, **f**). Scale bars = 3.5 μm.

Mineralization of ^13^C-biphenyl was expected to be accompanied by assimilation of ^13^C by biphenyl oxidizers. To test this hypothesis, subsamples of the labeling assays collected at 0, 24, 48, and 96 h of incubation were analyzed by nanoSIMS. Individual cells were defined based on secondary ion images of ^12^C^14^N^−^ and ^13^C^14^N^−^. The ^13^C abundance was determined for each cell. After 96 h of incubation, cells showing substantial enrichment in ^13^C were observed in all four soils (Fig. 2 and Supplementary Figs. 3–7). Most showed ^13^C enrichment close to the substrate labeling ratio (10 atom%) at the single-cell level. The relatively short incubation time and the high labeling ratio indicated that labeled cells were most likely primary biphenyl oxidizers, although the presence of secondary consumers feeding on excreted metabolites cannot be presently excluded. Biphenyl oxidizers are known to metabolize biphenyl to benzoate or other intermediates, which can be excreted and serve as a growth substrate for other members of the microbial communities [57, 58]. The variations in ^13^C abundance among individual cells could be caused by metabolic heterogeneity of primary biphenyl oxidizers [59, 60], or by poor substrate availability in the soil matrix. Alternatively, the less ^13^C-enriched cells may represent secondary consumers. Quantitatively, the cells enriched in ^13^C accounted for approximately 11%, 3%, 15%, and 8% of the total cell number of soils A, B, C, and D, respectively.

Carbon assimilation rate (*F*_*c*_) was calculated using the cellular ^13^C^14^N^−^/^12^C^14^N^−^ ratios and cell volumes calculated for regions of interest of nanoSIMS images [37]. For the calculation, we considered for all individual cells an average carbon density of 93.4 fg × μm^−3^ (average of several *Proteobacteria* isolates [61, 62]), since *Proteobacteria* was the most abundant microbial group in all tested soils. The cells showing enrichment in ^13^C have assimilation rates up to 0.61 fg cell^−1^ h^−1^ for soil A, 0.40 fg cell^−1^ h^−1^ for soil B, 2.9 fg cell^−1^ h^−1^ for soil C, and 0.96 fg cell^−1^ h^−1^ for soil D. To our knowledge, this is the first study reporting carbon assimilation rates for single cells of hydrocarbon-degrading microorganisms directly in soil environments.

### Phylogenetic identification of biphenyl degraders using protein-SIP

Since microbial communities of all soils were highly diverse, a FISH-based phylogenetic identification of the ^13^C-labeled cells via a correlative FISH-SIMS analysis was not possible. Instead, we opted for a high-throughput protein-SIP–metagenomics approach. For this, we used microcosm subsamples collected at 96 h incubation with labeled biphenyl since these showed the highest rates of substrate assimilation (Fig. 2 and Supplementary Fig. 7).

**Fig. 3:**
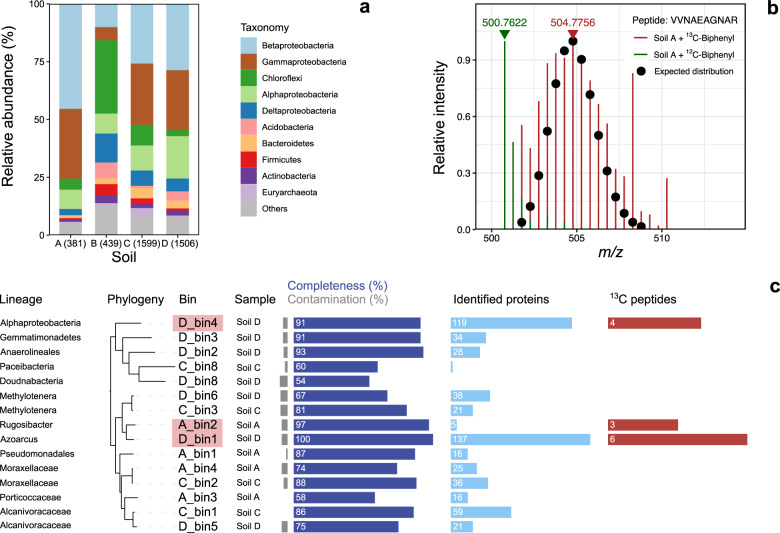
Identification of biphenyl-degrading microorganisms in soils using SIP-coupled metaproteomics. **a** Phylogenetic classification of identified proteins in four soil microcosms after 96-h incubation with biphenyl. The number in parenthesis indicates the number of identified proteins in the different soil samples. **b** Mass spectra of doubly charged peptide VVNAEAGNAR (PAH dioxygenase alpha subunit) in ^13^C (red lines) and ^12^C (green lines) microcosms. Black circles represent the best fit of the theoretical isotopic patterns. **c** Overview of medium-quality to high-quality MAGs obtained in this study. Phylogenetic classification, tree, and soil of origin for each MAG are shown on the left. Phylogeny of MAGs was reconstructed based on a concatenated alignment of 120 bacterial marker genes. Bars indicated the completeness, contamination, number of identified proteins, and number of detected ^13^C-peptides for each MAG. MAGs encoding ^13^C-peptides are shaded by pink boxes.

Metagenome sequencing of each sample was conducted to an average depth of 2.8 million reads. For soils A, B, C, and D de novo assembly yielded 274,602, 219,108, 554,918, and 236,566 scaffolds, respectively. Predicted open reading frames were compiled as soil-specific databases for global metaproteomic analyses. Soil proteins extracted from microcosms with labeled and unlabeled biphenyl were analyzed in parallel. Querying mass spectra against the protein databases resulted in the identification of 345, 410, 1070, and 1986 peptides, from 381, 349, 1599, and 1506 proteins for soil A, B, C, and D, respectively. The most frequently detected proteins were affiliated to *Proteobacteria*, *Chloroflexi*, *Acidobacteria*, and *Bacteroidetes* (Fig. 3a), consistent with taxa abundance revealed by 16S rRNA amplicon sequencing (Fig. 1a).

For the identified proteins, we analyzed the ^13^C incorporation level by comparing mass spectra of identified peptides from ^13^C-labeled vs. unlabeled microcosms. As an example, in soil A the peptide VVNAEAGNAR which is part of the PAH dioxygenase large subunit, showed a shift of the most abundant isotopomer from *m/z* of 500.7622 (*z* = 2) to 504.7756 (*z* = 2), accompanied by a Gaussian-like shaped isotopic pattern (Fig. 3b). The extent of the mass shift represented a relative isotope abundance (RIA) of 18.4%, close to the ^13^C abundance of the labeled substrate (20 atom% for protein-SIP). These results demonstrated ^13^C assimilation into proteins and suggested that the incorporation was achieved via direct substrate utilization, and not indirectly through a food web in which the label would be diluted. A similar ^13^C incorporation pattern was observed for 5 and 11 peptides in soils A and D (Supplementary Figs. 8–10), whereas no ^13^C-labeled peptides were detected in soils B and C. Possible reasons include the stringency of the statistical approach, low abundance of biphenyl degraders (i.e., in soil B), high complexity of the microbial community (i.e., soil C) or moderate recovery of soil proteins (soils B and C).

Phylogenetic classification of potential biphenyl degraders was achieved by mapping the ^13^C-labeled peptides to metagenome-assembled genomes (MAGs). Binning of the four assembled metagenomes resulted in a total of 15 medium to high-quality MAGs (>50% completeness and <10% contamination) comprising taxonomically distinct members of *Patescibacteria*, *Doudnabacteria*, *Gemmatimonadetes*, and *Proteobacteria* (Fig. 3c and Supplementary Tables 5–6). An average of 40 proteins was identified across all 15 MAGs, demonstrating their viability and activity in soils (Fig. 3c). Of these, the MAGs classified as *Alphaproteobacteria* (D_bin4) and *Azoarcus* (D_bin1) encoded 4 and, respectively, 6 uniquely mapped ^13^C-labeled peptides, indicating active biphenyl assimilation (Fig. 3c). A third MAG classified as uncultured *Rugosibacter* (A_bin2) encoded three unique ^13^C-labeled peptides. However, since for this MAG only a total of 5 unique proteins were identified (Fig. 3c), its assignment as active biphenyl oxidizer is tentative.

### Biphenyl degradation pathway in SIP-identified biphenyl-degraders

**Fig. 4:**
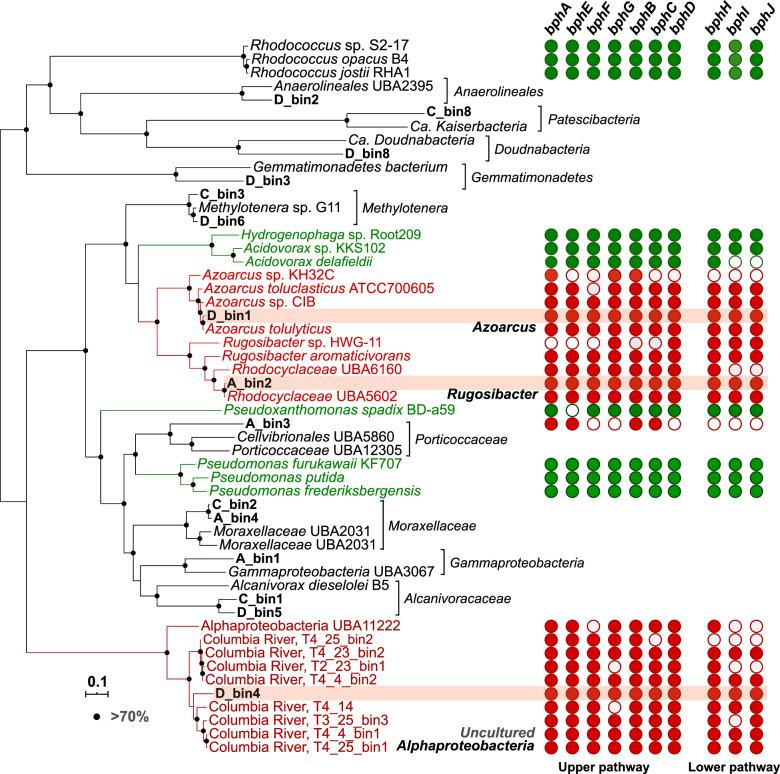
Phylogenetic placement of 15 MAGs recovered from soils. Tree clades containing ^13^C-labeled MAGs detected in this study are shown in red. Known biphenyl-degrading species are highlighted in green. The genomic tree was built from the concatenation of 120 bacterial marker genes. Filled circles on tree nodes indicated bootstrap values >70%. Scale bar, amino acid substitutions per site. The distribution of *bph* genes is shown in the right panel. Filled and empty circles represent the presence or absence of *bph* genes. The gene distribution is not shown for lineages without detectable *bph* homologs. Abbreviations: *bphA*: biphenyl dioxygenase subunit alpha; *bphE*: biphenyl dioxygenase subunit beta; *bphF*: biphenyl dioxygenase ferredoxin component; *bphG*: biphenyl dioxygenase system ferredoxin/NAD^+^ reductase component; *bphB*: *cis*-2,3-dihydrobiphenyl-2,3-diol dehydrogenase; *bphC*: 2,3-dihydroxybiphenyl 1,2-dioxygenase; *bphD*: 2-hydroxy-6-oxo-6-phenylhexa-2,4-dienoate hydrolase; *bphI*: 4-hydroxy-2-oxovalerate aldolase; *bphH*: 2-oxopent-4-enoate hydratase; *bphJ*: acetaldehyde dehydrogenase.

**Fig. 5:**
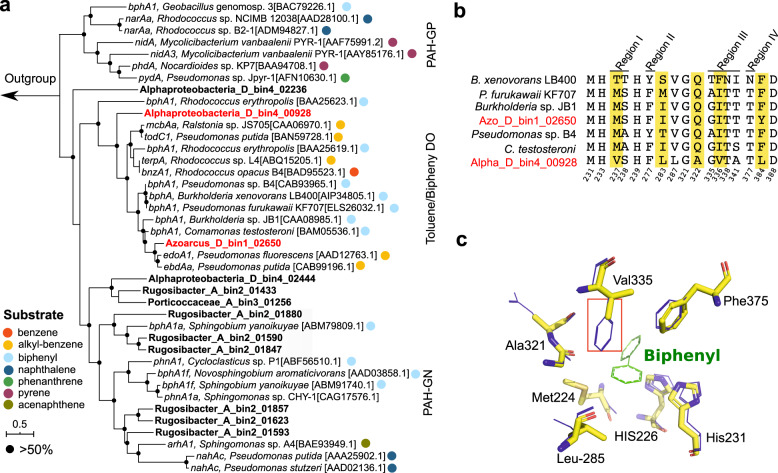
Phylogeny and homology modeling of the alpha subunit of biphenyl dioxygenase (BphA). **a** BphA homologs from metagenomic bins are shown in bold font, and those affiliated to toluene/biphenyl dioxygenase (DO) are highlighted in red color. Filled circles on the tree branches indicated the bootstrap values >50%. The scale bar represents the number of substitutions per site. PAH-GN (GP): PAH dioxygenase from Gram-negative (Gram-positive) bacteria; T/B DO: toluene/biphenyl dioxygenase. **b** Sequence alignment of BphA from A_bin2, D_bin4, and well-known biphenyl degraders. Sequence regions that influenced the PCB congener preference and regiospecificity of biphenyl dioxygenase, are displayed [79, 80]. *B. xenovorans*: *Burkholderia xenovorans*; *P. furukawaii*: *Pseudomonas furukawaii*; *C. testosteroni*: *Comamonas testosteroni*. The amino acid numbering corresponds to BphA of the *B. xenovorans* LB400 sequence. **c**, Modeled substrate binding pocket in the BphA of D_bin4. The predicted BphA structure (yellow sticks) was superimposed on *B. xenovorans* BphA (blue wireline). The biphenyl molecule is shown as green lines.

Each of the three MAGs identified by protein-SIP provided further genomic evidence for the utilization of biphenyl (Fig. 4). All three MAGs contained genes encoding homologs of biphenyl dioxygenase (BPDO), the first enzyme of the biphenyl oxidation pathway [16]. To confirm the relatedness of these genes to known BPDO, we constructed a phylogenetic tree using proteins of the BPDO catalytic subunit (BphA). This analysis revealed that the BphAs from D_bin1 and D_bin4 belonged to a toluene/biphenyl dioxygenase (T/B DO) clade closely related to those of biphenyl-degrading *Burkholderia xenovorans* LB400 and *Pseudomonas furukawaii* KF707 (Fig. 5a) [63, 64]. In contrast, BphAs encoded by A_bin2 exhibited higher sequence similarity with biochemically characterized BphA from *Sphingobium yanoikuyae* within the PAH-GN clade (Fig. 5a) [65]. In addition to *bphA* genes, D_bin4 and A_bin2 contained multiple copies of paralogs that were genetically distant and phylogenetically distinct from known BPDOs (Fig. 5a). These genes could possibly be responsible for the activation of aromatic compounds other than biphenyl (e.g., naphthalene).

**Fig. 6:**
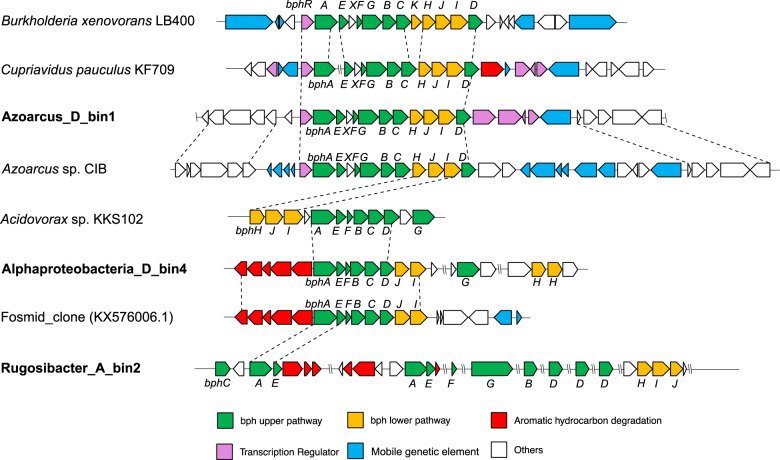
Alignment of genomic region(s) of three ^13^C-labeled MAGs encoding *bph* genes with corresponding regions of reference genomes. Conserved genomic regions with similar gene organization are highlighted using dashed lines.

Furthermore, all three MAGs contained genes of the biphenyl upper and lower pathways [16]. Specifically, these included genes encoding *cis*-2,3-dihydrobiphenyl-2,3-diol dehydrogenase (BphB), 2,3-dihydroxybiphenyl 1,2-dioxygenase (BphC), and 2-hydroxy-6-oxo-6-phenylhexa-2,4-dienoate hydrolase (BphD) for the upper pathway, and genes coding for 4-hydroxy-2-oxovalerate aldolase (BphI), 2-oxopent-4-enoate hydratase (BphH), and acetaldehyde dehydrogenase (BphJ) for the lower pathway (Figs. 4 and 6; Supplementary Table 7). In D_bin1 and D_bin4, genes for the upper and lower pathways were present in operons showing similar synteny with that of *B. xenovorans* LB400 [66]. In the *Rugosibacter* A_bin2, synteny was absent, the same set of genes being located at different genomic regions (Fig. 6).

The biphenyl upper and lower pathways yield eventually benzoate, which can be further metabolized to TCA intermediates via two different pathways. D_bin4 and A_bin2 encoded benzoate 1,2-dixoygenase (BenAB) and/or catechol 2,3-dioxygenase (DmpB) (Supplementary Table 8), suggesting the corresponding microorganisms were able to oxidize benzoate via the catechol meta-cleavage pathway [67]. By contrast, D_bin1 appeared to oxidize benzoate via the benzoyl-CoA pathway, as shown by the presence of genes coding benzoate coenzyme A ligase (BadA) and benzoyl-CoA 2,3-epoxidase (BoxAB) [68]. Finally, all three MAGs encode complete TCA cycle enzymes and multiple aerobic respiration complexes that could enable them to completely oxidize biphenyl to CO_2_ and conserve energy via oxygen respiration, consistent with a strict or facultative aerobic lifestyle (Supplementary Tables 9–10). These findings further consolidated the identification of the uncultured *Alphaproteobacteria* (D_bin4) and *Azoarcus* (D_bin1) as active biphenyl-degrading microorganisms in the tested soils. Although the uncultured *Rugosibacter* A_bin2 encodes two BphAs highly similar to biochemically characterized enzymes and all genes required for complete biphenyl oxidation, it lacks the syntenic organization of the *bph* genes typical of biphenyl oxidizers. Its assignment as biphenyl oxidizer remains therefore cautious.

## Discussion

Here we explored the largely uncultured soil microbiota mediating organic pollutants degradation via an experimental framework that integrated stable isotope probing with single-cell analysis and isotope-specific molecular biology approaches. Biphenyl-assimilating microorganisms were visualized directly in soils via nanoSIMS chemical imaging. With this approach, we determined both the abundance of active biphenyl oxidizers in different soils and their rates of biphenyl assimilation (Fig. 2). Taxonomic identification of labeled cells based on protein-SIP showed an obvious difference between biphenyl oxidizers active under near in situ conditions and species enriched via repeated sub-cultivation. In contrast to well-known biphenyl degraders yielded by the cultivation-dependent approach, our labeling approach revealed previously unrecognized functions of uncultured yet ecologically relevant species. The clade UBA11222 represents such an uncultivated *Alphaproteobacteria* lineage whose physiological functions remained so far undocumented, despite their broad distribution in terrestrial and marine environments associated with organic contaminants (Supplementary Fig. 11) [7, 69]. Here we provided both physiological and genomic evidence that members of this clade are able to derive carbon and energy from the oxidation of biphenyl (Figs. 2 and 3). Considering that aromatic hydrocarbon oxidation pathways often have extended substrate spectra and that biphenyl-oxidizing microorganisms can often co-metabolize PCBs, members of this clade may have broader environmental implications in biodegradation processes. In addition, the combination of activity probing and genomic reconstruction indicated greater biodegradation capacities for known hydrocarbon-degrading bacteria. Strains of *Azoarcus* and *Rugosibacter* have been frequently retrieved as toluene and pyrene degraders, respectively [70, 71]. Their in situ activities towards biphenyl, as revealed (*Azoarcus*) or suggested (*Rugosibacter*) here, thus indicate a broader substrate range and higher metabolic versatility than previously recognized.

Genome analysis of taxa that we identified as active hydrocarbon oxidizers provided novel mechanistic insights regarding biodegradation processes in nature. Biphenyl dioxygenase (BPDO), the key enzyme for biodegradation of biphenyl and its analogs (i.e., PCBs), was detected in all three SIP-identified bacteria. The BPDO from the clade UBA11222 apparently belonged to an offset cluster within biphenyl/toluene dioxygenase family [72], with clear separation from those of cultivated biphenyl-utilizing bacteria (Fig. 5a). Their catalytic subunit (BphA) exhibited distinct sequence patterns of amino acids that were known to influence enzyme-substrate specificity and regiospecificity (Fig. 5b). Consistently, structure modeling of BphA from UBA11222 based on the available BphA crystal structure (*Burkholderia xenovorans* LB400) revealed differences between the amino acid sequences of the substrate-binding pocket (Fig. 5c). Replacement of an aromatic amino acid by an aliphatic residue may allow UBA11222 BPDO to accommodate different sets of PCB substrates. These results extend the genetic diversity of *bphA* in soil environments and imply a divergent function of BPDO in uncultured biphenyl-degrading microorganisms compared to cultivated counterparts. In two SIP-identified MAGs from soil D, *bphA* was found at genomic regions enriched in mobile genetic elements (MGEs), including those encoding transposes and integrase (Fig. 6). This concurs with previous findings of close genetic associations between MGEs and catabolic genes in isolated PCB-degrading microorganisms [16]. These observations suggest that horizontal gene transfer (HGT) mediated by MGEs may play a major role in the in situ dissemination of catabolic pathways, potentially allowing rapid adaptation of microbial communities to organic pollutants [73]. In addition, metabolic capacities of complete biphenyl oxidation were apparently conserved across phylogenetically distinct MAGs retrieved from the same soil, suggesting some functional plasticity of the soil microbial communities. This appears distinct from oil-degrading microbial communities of oil-contaminated environments, where the main catabolic pathways are partitioned among individual community members, and complete oxidation of hydrocarbons is achieved by complex coordination between microorganisms carrying out non-redundant reactions [74]. This disparity reflects diverse modes of microbial biodegradation networks in the environment [75].

All the ^13^C-labeled peptides identified here were mapped to MAGs with a genetic makeup indicating a potential for complete oxidation of biphenyl. However, nanoSIMS analyses showed a small number of cells becoming lightly enriched in ^13^C during the later incubation times (Fig. 2, Supplementary Figs. 3–7). These could represent secondary consumers feeding on metabolites excreted by the primary biphenyl oxidizers. The most likely reasons secondary consumers were not apparent in the protein-SIP analyses were their relatively low abundance and low ^13^C enrichment level. Future studies employing time-resolved ecophysiology probing over longer incubation times are needed to resolve metabolic networks involved in the degradation of biphenyl or other contaminants and to reveal the identity of secondary consumers.

Our findings have two kinds of implications. On the one hand, taxonomic identification of resident clades and measurement of their near-in-situ hydrocarbon uptake rates may assist to devise tailored bioremediation strategies. We showed that it was uncultured taxa distinct from those retrieved by enrichment approaches that responded readily to biphenyl additions under near in situ conditions. For example, in slurries of soil D, we identified *Azoarcus*, while cultivation yielded *Pseudomonas* and *Rhodococcus*. *Azoarcus* and the related *Aromatoleum* and *Thauera* genera are best known for their metabolic plasticity and ability to oxidize aromatic hydrocarbons under nitrate-reducing conditions (Supplementary Fig. 12) [76]. Therefore, in environments like soil D, a more effective bioremediation strategy might be to enhance the natural attenuation potential for example by stimulating nitrate reduction, instead of the more common practices of aeration or injection of oxygen [77, 78].

On the other hand, the wealth of metabolic pathways retrieved during the past decade via environmental sequencing is in dire need of direct evidence for their functionality [10, 11]. Extending the approach used here to other substrates and environments may allow testing of predicted ecophysiologies, uncover in situ metabolic networks, or identify metabolically active microorganisms, which, as shown here, could be rather different than those obtained via long-time cultivation. Combined with rapid innovations in cultivation strategies [4], direct in situ or near in situ ecophysiology probing is one of the awaited approaches to expand our knowledge of the metabolic potential and ecological roles of members of the uncultured majority.

## Supplementary information

Supplementary Information

## Data Availability

Sequencing data are archived in the NCBI database under BioProject number PRJNA692092, including the metagenome and 16 S rRNA gene amplicon datasets for soil A (SAMN17313532), B (SAMN17313578), C (SAMN17313580), and D (SAMN17313590). Metagenome-assembled genomes (MAGs) of uncultured biphenyl-degrading bacteria, including Rugosibacter_A_bin2 (SAMN17315487), Azoarcus_D_bin1 (SAMN17315485), and Alphaproteobacteria_D_bin4 (SAMN17315486), have been submitted to NCBI under the same BioProject. The proteomics dataset has been deposited with the ProteomeXchange Consortium identifier PXD023646. All other data are available in the paper or the Supplementary Information.
