# Supplementary material for: Novel clades of soil biphenyl degraders revealed by integrating isotope probing, multi-omics, and single-cell analyses
Source: ISME J. 2021 Jun 11;15(12):3508–21. doi: 10.1038/s41396-021-01022-9 (PMC8630052; doi:10.1038/s41396-021-01022-9)
Supplement: Supplementary file 1 — Supplementary Information [file 41396_2021_1022_MOESM1_ESM.docx]

**Novel clades of soil biphenyl degraders revealed by integrating isotope probing, multi-omics, and single-cell analyses**

Song-Can Chen^1^, Rohit Budhraja^1^, Lorenz Adrian^2,3^, Federica Calabrese^1^, Hryhoriy Stryhanyuk^1^, Niculina Musat^1^, Hans-Hermann Richnow^1^, Gui-Lan Duan^4^, Yong-Guan Zhu^4,5,*^, Florin Musat^1,*^

1 Department of Isotope Biogeochemistry, Helmholtz Centre for Environmental Research – UFZ, 04318 Leipzig, Germany

2 Department Environmental Biotechnology, Helmholtz Centre for Environmental Research – UFZ, Leipzig, Germany

3 Chair of Geobiotechnology, Technische Universität Berlin, 13355 Berlin, Germany

4 State Key Lab of Urban and Regional Ecology, Research Center for Eco-Environmental Sciences, Chinese Academy of Sciences, Beijing 100085, China

5 Key Lab of Urban Environment and Health, Institute of Urban Environment, Chinese Academy of Sciences, Xiamen 361021, China

* Correspondence to: [florin.musat@ufz.de](mailto:florin.musat@ufz.de) and [ygzhu@rcees.ac.cn](mailto:ygzhu@rcees.ac.cn)

Table S1. The type and origin of the four soils used in this study, and their level of contamination with polychlorinated biphenyls (PCBs).

| Soil | Soil type | Latitude | Longitude | PCBs conc. (mg/kg) |
| --- | --- | --- | --- | --- |
| A | River sediment | 28°32'19"N | 121°22'41"E | 4.0 - 5.0 |
| B | Paddy soil | 28°32'28"N | 121°22'15"E | 0.032 - 0.93 |
| C | River sediment | 28°37'13"N | 121°24'54"E | 2.0 - 3.0 |
| D | Upland soil | 28°30'32"N | 121°21'49"E | 4.0 - 5.0 |

Table S2. Alpha diversity of microbial communities in the four soils investigated. Metrics of alpha diversity, including Shannon index, Chao1, Simpson and Simpson’s evenness measure E were calculated from the OTU table created by SilvaNGS.

| Soil | # reads | # OTUs^1^ | Shannon | Chao1 | Simpson | Simpson_E^2^ |
| --- | --- | --- | --- | --- | --- | --- |
| A | 36653 | 18188 | 13.20 | 69023.96 | 1.00 | 0.12 |
| B | 79818 | 29517 | 13.61 | 88761.85 | 1.00 | 0.11 |
| C | 149527 | 49167 | 14.12 | 154060.28 | 1.00 | 0.08 |
| D | 106941 | 35120 | 13.47 | 131315.52 | 1.00 | 0.06 |

^1^: observed OTUs reported by SilvaNGS

^2^: Simpson’s evenness measure E.

Table S3. Total number of cells analyzed by nanoSIMS and the number of cells showing ^13^C-enrichment in the four soils at four sampling times.

| Soil | Incubation time | # Cells analyzed in total | # Cells showing ^13^C enrichment | Relative abundance of ^13^C-enriched cells |
| --- | --- | --- | --- | --- |
| A | 0 h^1^ | 27 | 0 | 0.0% |
|  | 24 h^2^ | 117 | 0 | 0.0% |
|  | 48 h^2^ | 127 | 9 | 7.1% |
|  | 96 h^3^ | 97 | 12 | 12.4% |
| B | 0 h^1^ | 56 | 0 | 0.0% |
|  | 24 h^2^ | 109 | 0 | 0.0% |
|  | 48 h^2^ | 129 | 0 | 0.0% |
|  | 96 h^3^ | 172 | 6 | 3.5% |
| C | 0 h^1^ | 69 | 0 | 0.0% |
|  | 24 h^2^ | 182 | 3 | 1.6% |
|  | 48 h^2^ | 160 | 36 | 22.5% |
|  | 96 h^3^ | 176 | 24 | 13.6% |
| D | 0 h^1^ | 48 | 0 | 0.0% |
|  | 24 h^2^ | 134 | 2 | 1.5% |
|  | 48 h^2^ | 175 | 1 | 0.6% |
|  | 96 h^3^ | 202 | 17 | 8.4% |

^1^ The cells were collected from 1 field of view of nanoSIMS analyses

^2^ The cells were collected from 2 fields of view of nanoSIMS analyses

^3^ The cells were collected from 3 fields of view of nanoSIMS analyses

Table S4. Protein sequences of *bph* genes used to query against MAGs. BphAs in each species are highlighted in bold.

| Species | Protein sequences encoded by *bph* genes^1^ | Reference |
| --- | --- | --- |
| *Acidovorax* sp. KKS102 | BphH (WP_015014858.1), BphJ (WP_015014859.1), BphI (WP_015014860.1), BphX (WP_015014861.1), **BphA (WP_085944663.1)**, BphE (WP_043566691.1), BphF (WP_015014864.1), BphB (WP_015014865.1), BphC (WP_015014866.1), BphD (WP_015014867.1), BphG (WP_168162369.1) | [1] |
| *Alcanivorax* sp. HA03 | **BphA1 (AFD18268.1)**, BphA2 (AFD18269.1), BphA3 (AFD18271.1), BphA4 (AFD18272.1) | [2] |
| *Bacillus* sp. JF8 | BphR (BAC79224), BphD (BAC79225), **BphA1 (BAC79226)**, BphA2 (BAC79227), BphB (BAC79228) | [3] |
| *Burkholderia* sp. JB1 | **BphA1 (CAA08985.1)** | [2] |
| *Burkholderia xenovorans* LB400 | BphR (WP_011494300.1), **BphA (WP_011494299.1)**, BphE (WP_040123616.1), BphX (WP_003451002.1), BphF (WP_011494297.1), BphG (WP_003450997.1), BphB (WP_011494296.1), BphC (WP_011494295.1), BphK (WP_003450988.1), BphH (WP_003450985.1), BphJ (WP_003450975.1), BphI (WP_003450974.1), BphD (WP_011494293.1) | [4] |
| *Comamonas testosterone* | **BphA1 (BAC01052)**, BphA2 (BAC01053), BphA3 (BAC01054), BphB (BAC01055), BphC (BAC01056), BphD (BAC01057) | [5] |
| *Comamonas testosteroni* YU14-111 | BphE (BAM05532.1), BphG (BAM05533.1), BphF (BAM05534.1), BphX (BAM05535.1), **BphA1 (BAM05536.1)**, BphA2 (BAM05537.1), BphA3 (BAM05538.1), BphB (BAM05539.1), BphC (BAM05540.1), BphD (BAM05541.1), BphA4 (BCA43067.1) | [2] |
| *Cupriavidus pauculus* | BphD (WP_061959573.1), BphI (WP_061959574.1), BphJ (WP_061959575.1), BphH (WP_061959576.1), BphC (WP_061959583.1), BphB (WP_061959577.1), BphG (WP_061959578.1), BphF (WP_084004241.1), BphX (WP_061959579.1), BphE (WP_061959580.1), **BphA (WP_061959585.1)**, BphR (WP_084004242.1) | [6] |
| *Cupriavidus* sp. SK-4 | BphR (AFC89841.1), **BphA1 (AFC89842.1)**, BphA2 (AFC89843.1), BphA3 (AFC89845.1), BphA4 (AFC89846.1), BphB (AFC89847.1), BphC (AFC89848.1), BphK (AFC89849.1), BphH (AFC89850.1), BphJ (AFC89851.1), BphI (AFC89852.1), BphD (AFC89853.1) | [7] |
| *Janibacter* sp. TYM3221 | **BphAa (BAK52803.1)**, BphAb (BAK52804.1), BphAc (BAK52805.1), BphD (BAM76235.1), BphAa (BAM76236.1), BphAb (BAM76237.1), BphAc (BAM76238.1), BphAd (BAM76239.1), BphB (BAM76240.1), BphC (BAM76241.1), BphS (BAM76242.1), BphT (BAM76243.1) | [8] |
| *Pandoraea pnomenusa* strain B-356 | **BphA (AAC44526.1)**, BphE (AAC44527.1), BphX (AAC44528.1), BphF (AAC44529.1), BphB (AAC44530.1) | [9] |
| *Pseudomonas furukawaii* | BphR (WP_036992482.1), **BphA (WP_036992472.1)**, BphE (WP_003451003.1), BphX (WP_003451002.1), BphF (WP_011494297.1), BphG (WP_003450997.1), BphB (WP_036992465.1), BphC (WP_029380758.1), BphK (WP_003450988.1), BphH (WP_003450985.1), BphJ (WP_003450975.1), BphI (WP_003450974.1), BphD (WP_003450973.1) | [10] |
| *Pseudomonas pseudoalcaligenes* KF707 | **BphA1 (AAA25743.1)**, BphA2 (AAA25744.1), BphA3 (AAA25746.1), BphA4 (AAA25747.1), BphB (AAA25748.1), BphC (AAA25749.1) | [11] |
| *Pseudomonas putida* strain B6-2 | **BphA1 (ACN62349.1)**, BphA2 (ACN62350.1), BphA3 (ACN62352.1), BphA4 (ACN62353.1), BphB (ACN62354.1), BphC (ACN62355.1) | [12] |
| *Pseudomonas* sp. B4 | **BphA1 (CAB93965.1)**, BphA2 (CAB93966.1), BphA3 (CAB93968.1), BphA4 (CAB93969.1) | [13] |
| *Pseudomonas* sp. Cam-1 | **BphA (AAK14781.1)**, BphE (AAK14782.1), BphF (AAK14784.1), BphG (AAK14785.1), BphB (AAK14786.1), BphC (AAK14787.1) | [14] |
| *Pseudomonas* sp. KKS102 | **BphA1 (BAA04137.1)**, BphA2 (BAA04138.1), BphA3 (BAA04139.1), BphB (BAA04140.1), BphC (BAA04141.1) | [1] |
| *Ralstonia oxalatica* | **BphA1 (CAD61140.1)**, BphA2 (CAD61141.1), BphA3 (CAD61142.1), BphB (CAD61143.1), BphC (CAD61144.1), BphD (CAD61145.1), BphA4 (CAD61147.1) | [15] |
| *Rhodococcus erythropolis* | BphB (BAF48500.1), BphC (BAF48501.1), **BphA1 (BAF48503.1)**, BphA2 (BAF48504.1), BphA3 (BAF48507.1), BphA4 (BAF48508.1), BphD (BAF48509.1) | [16] |
| *Rhodococcus erythropolis* TA421 | **BphA1 (BAA25619.1)**, BphA2 (BAA25620.1), BphA3 (BAA25621.1), BphA4 (BAA25622.1) | [17] |
| *Rhodococcus globerulus* | **BphA1 (CAA56346.1)**, BphA2 (CAA56347.1), BphA3 (CAA56348.1), BphA4 (CAA56349.1) | [18] |
| *Rhodococcus rhodochrous* | BphB (BAF48488.1), BphC8 (BAF48489.1), **BphA1 (BAF48491.1)**, BphA2 (BAF48492.1), BphA3 (BAF48495.1), BphA4 (BAF48496.1), BphD (BAF48497.1) | [16] |
| *Rhodococcus* sp. HA99 | BphB (BAF48517.1), BphC (BAF48518.1), **BphA1 (BAF48520.1)**, BphA2 (BAF48521.1), BphA3 (BAF48524.1), BphA4 (BAF48525.1), BphD (BAF48526.1) | [16] |
| *Rhodococcus* sp. R04 | BphB (ABD65914), BphC (ABD65915), **BphA1 (ABD65916)**, BphA2 (ABD65917), BphA3 (ABD65918), BphA4 (ABD65919), BphD (ABD65920) | [19] |
| *Rhodococcus* sp. strain RHA1 | **BphA1 (BAA06868.1)**, BphA2 (BAA06869.1), BphA3 (BAA06870.1), BphA4 (BAA06871.1), BphC (BAA06872.1), BphB (BAA06873.1) | [20] |
| *Sphingobium yanoikuyae* B1 | **BphA1f (ABM91740.1)**, BphA2f (ABM91741.1), BphA2b (ABM79806.1), BphA2a (ABM79808.1), BphA1a (ABM79809.1), BphR (ABM79810.1), BphB (ABM79802.1) | [21] |
| *Sphingomonas aromaticivorans* plasmid pNL1 | BphA1b (AAD03982.1), **BphA1a (AAD03980.1)**, BphA1f (AAD03858.1) | [22] |

^1^ NCBI GenBank accession for the protein sequence was placed in parentheses.

Table S5. Taxonomic affiliation and assembly statistics of 15 metagenome-assembled genomes (completeness >50% and contamination <10%) from the four PCB-contaminated soils (namely A, B, C, and D). Completeness and contamination of MAGs were estimated by checkM.

| MAGs | Completeness  (%) | Contamination  (%) | GC (%) | Predicted genes (#) | Total length (Mbp) | GTDB classification | | | | |
| --- | --- | --- | --- | --- | --- | --- | --- | --- | --- | --- |
|  |  |  |  |  |  | Class | Order | Family | Genus |  |
| A_bin1 | 86.84 | 9.74 | 49 | 2937 | 2.64 | *Gammaproteobacteria* | *Pseudomonadales* | *UBA3067* | *UBA3067* |  |
| A_bin2 | 96.88 | 0.62 | 57 | 2494 | 2.64 | *Gammaproteobacteria* | *Burkholderiales* | *Rhodocyclaceae* | *Rugosibacter* |  |
| A_bin3 | 57.88 | 11.43 | 64 | 2888 | 2.24 | *Gammaproteobacteria* | *Pseudomonadales* | *Porticoccaceae* |  |  |
| A_bin4 | 73.50 | 3.47 | 57 | 2199 | 1.98 | *Gammaproteobacteria* | *Pseudomonadales* | *Moraxellaceae* |  |  |
| C_bin1 | 56.03 | 3.45 | 62 | 1623 | 1.57 | *Gammaproteobacteria* | *Pseudomonadales* | *Alcanivoracaceae* |  |  |
| C_bin2 | 88.36 | 5.75 | 57 | 2638 | 2.48 | *Gammaproteobacteria* | *Pseudomonadales* | *Moraxellaceae* |  |  |
| C_bin3 | 80.97 | 1.01 | 45 | 2789 | 2.39 | *Gammaproteobacteria* | *Burkholderiales* | *Methylophilaceae* |  |  |
| C_bin8 | 59.74 | 7.64 | 40 | 926 | 0.86 | *Paceibacteria* | *UBA9983_A* | *UBA2163* |  |  |
| D_bin1 | 99.69 | 3.26 | 66 | 5456 | 5.74 | *Gammaproteobacteria* | *Burkholderiales* | *Rhodocyclaceae* | *Azoarcus_A* |  |
| D_bin2 | 93.39 | 5.68 | 50 | 3544 | 3.31 | *Anaerolineae* | *Anaerolineales* | *envOPS12* | *OLB14* |  |
| D_bin3 | 91.08 | 4.96 | 69 | 3303 | 2.95 | *Gemmatimonadetes* | *Gemmatimonadales* | *GWC2-71-9* |  |  |
| D_bin4 | 92.33 | 2.60 | 58 | 3160 | 2.96 | *Alphaproteobacteria* | *UBA11222* | *UBA11222* |  |  |
| D_bin5 | 75.18 | 3.29 | 61 | 2384 | 2.03 | *Gammaproteobacteria* | *Pseudomonadales* | *Alcanivoracaceae* |  |  |
| D_bin6 | 66.93 | 1.18 | 50 | 1860 | 1.64 | *Gammaproteobacteria* | *Burkholderiales* | *Methylophilaceae* | *Methylotenera* |  |
| D_bin8 | 54.24 | 0.29 | 38 | 652 | 0.48 | *Doudnabacteria* | *UBA920* |  |  |  |

Table S6. Reads abundance of the 15 MAGs retrieved from the four soils. Reads mapping was performed using BBMap (v38.51) with individual MAGs reference sequence database. The minimum alignment identity (minid) parameter of BBmap was set as 0.9.

| MAGs | Soil A | Soil B | Soil C | Soil D |
| --- | --- | --- | --- | --- |
| Pseudomonadales_A_bin1 | 1.25% | 0.00% | 0.01% | 0.00% |
| Rugosibacter_A_bin2 | 3.52% | 0.00% | 0.04% | 0.01% |
| Porticoccaceae_A_bin3 | 0.91% | 0.01% | 0.42% | 0.12% |
| Moraxellaceae_A_bin4 | 1.08% | 0.00% | 0.57% | 0.00% |
| Alcanivoracaceae_C_bin1 | 0.20% | 0.00% | 0.51% | 0.59% |
| Moraxellaceae_C_bin2 | 1.21% | 0.00% | 0.80% | 0.01% |
| Methylotenera_C_bin3 | 0.22% | 0.00% | 0.59% | 0.18% |
| Paceibacteria_C_bin8 | 0.00% | 0.00% | 0.63% | 0.00% |
| Azoarcus_D_bin1 | 0.01% | 0.00% | 0.01% | 6.14% |
| Anaerolineales_D_bin2 | 0.00% | 0.00% | 0.01% | 3.00% |
| Gemmatimonadales_D_bin3 | 0.00% | 0.00% | 0.00% | 1.91% |
| Alphaproteobacteria_D_bin4 | 0.01% | 0.00% | 0.05% | 2.39% |
| Alcanivoracaceae_D_bin5 | 0.15% | 0.00% | 0.56% | 0.99% |
| Methylotenera_D_bin6 | 0.01% | 0.02% | 0.02% | 0.81% |
| Doudnabacteria_D_bin8 | 0.00% | 0.00% | 0.00% | 0.18% |

Table S7. Genes encoding upper and lower pathways for biphenyl biodegradation in A_bin2 (*Rugosibacter*), D_bin1 (*Azoarcus*), and D_bin4 (*Alphaproteobacteria* UBA11222).

| Gene | Feature | Locus tag | Amino acids |
| --- | --- | --- | --- |
| Upper pathway | |  |  |
| *bphA* | Alkylbenzene/biphenyl dioxygenases, large subunit | A_bin2_01433 | 455 |
| *bphA* | Naphthalene/biphenyl dioxygenases, large subunit | A_bin2_01590 | 452 |
| *bphA* | Naphthalene/biphenyl dioxygenases, large subunit | A_bin2_01593 | 448 |
| *bphA* | Naphthalene/biphenyl dioxygenases, large subunit | A_bin2_01623 | 444 |
| *bphA* | Naphthalene/biphenyl dioxygenases, large subunit | A_bin2_01847 | 453 |
| *bphA* | Naphthalene/biphenyl dioxygenases, large subunit | A_bin2_01857 | 443 |
| *bphA* | Naphthalene/biphenyl dioxygenases, large subunit | A_bin2_01880 | 453 |
| *bphA* | Biphenyl dioxygenases, large subunit | D_bin1_02650 | 458 |
| *bphA* | Biphenyl dioxygenases, large subunit | D_bin4_00928 | 472 |
| *bphE* | Alkylbenzene/biphenyl dioxygenases, small subunit | A_bin2_01434 | 168 |
| *bphE* | Naphthalene/biphenyl dioxygenases, small subunit | A_bin2_01589 | 181 |
| *bphE* | Naphthalene/biphenyl dioxygenases, small subunit | A_bin2_01594 | 175 |
| *bphE* | Naphthalene/biphenyl dioxygenases, small subunit | A_bin2_01622 | 174 |
| *bphE* | Naphthalene/biphenyl dioxygenases, small subunit | A_bin2_01848 | 181 |
| *bphE* | Naphthalene/biphenyl dioxygenases, small subunit | A_bin2_01858 | 174 |
| *bphE* | Naphthalene/biphenyl dioxygenases, small subunit | A_bin2_01879 | 183 |
| *bphE* | Biphenyl dioxygenases, small subunit | D_bin1_02649 | 186 |
| *bphE* | Biphenyl dioxygenases, small subunit | D_bin4_00927 | 182 |
| *bphF* | Biphenyl dioxygenase, ferredoxin component | A_bin2_01629 | 109 |
| *bphF* | Biphenyl dioxygenase, ferredoxin component | D_bin1_02647 | 111 |
| *bphF* | Biphenyl dioxygenase, ferredoxin component | D_bin4_00926 | 107 |
| *bphG* | Biphenyl dioxygenase, ferredoxin/NAD+ reductase component | A_bin2_01027 | 831 |
| *bphG* | Biphenyl dioxygenase, ferredoxin/NAD+ reductase component | D_bin1_02646 | 413 |
| *bphG* | Biphenyl dioxygenase, ferredoxin/NAD+ reductase component | D_bin4_00482 | 407 |
| *bphB* | Cis-2,3-dihydrobiphenyl-2,3-diol dehydrogenase | A_bin2_01619 | 271 |
| *bphB* | Cis-2,3-dihydrobiphenyl-2,3-diol dehydrogenase | D_bin1_02645 | 285 |
| *bphB* | Cis-2,3-dihydrobiphenyl-2,3-diol dehydrogenase | D_bin4_00925 | 297 |
| *bphC* | 2,3-dihydroxybiphenyl 1,2-dioxygenase | A_bin2_01109 | 310 |
| *bphC* | 2,3-dihydroxybiphenyl 1,2-dioxygenase | A_bin2_01845 | 307 |
| *bphC* | 2,3-dihydroxybiphenyl 1,2-dioxygenase | D_bin1_02644 | 310 |
| *bphC* | 2,3-dihydroxybiphenyl 1,2-dioxygenase | D_bin4_00924 | 298 |
| *bphD* | 2-hydroxy-6-oxo-6-phenylhexa-2,4-dienoate hydrolase | A_bin2_00365 | 289 |
| *bphD* | 2-hydroxy-6-oxo-6-phenylhexa-2,4-dienoate hydrolase | A_bin2_01607 | 285 |
| *bphD* | 2-hydroxy-6-oxo-6-phenylhexa-2,4-dienoate hydrolase | A_bin2_02057 | 277 |
| *bphD* | 2-hydroxy-6-oxo-6-phenylhexa-2,4-dienoate hydrolase | D_bin1_02640 | 287 |
| *bphD* | 2-hydroxy-6-oxo-6-phenylhexa-2,4-dienoate hydrolase | D_bin4_00923 | 278 |
| Lower pathway | |  |  |
| *bphH* | 2-oxo-pent-4-enoate hydratase | A_bin2_00222 | 264 |
| *bphH* | 2-oxo-pent-4-enoate hydratase | A_bin2_00611 | 262 |
| *bphH* | 2-oxo-pent-4-enoate hydratase | A_bin2_00615 | 260 |
| *bphH* | 2-oxo-pent-4-enoate hydratase | D_bin1_02643 | 273 |
| *bphH* | 2-oxo-pent-4-enoate hydratase | D_bin4_00638 | 256 |
| *bphH* | 2-oxo-pent-4-enoate hydratase | D_bin4_00639 | 267 |
| *bphI* | 4-hydroxy-2-oxovalerate aldolase | A_bin2_00612 | 346 |
| *bphI* | 4-hydroxy-2-oxovalerate aldolase | D_bin1_02641 | 344 |
| *bphI* | 4-hydroxy-2-oxovalerate aldolase | D_bin4_00921 | 348 |
| *bphJ* | Acetaldehyde dehydrogenase | A_bin2_00613 | 306 |
| *bphJ* | Acetaldehyde dehydrogenase | D_bin1_02642 | 304 |
| *bphJ* | Acetaldehyde dehydrogenase | D_bin4_00922 | 313 |

Table S8. Genes encoding pathways for benzoate biodegradation in A_bin2 (*Rugosibacter*), D_bin1 (*Azoarcus*), and D_bin4 (*Alphaproteobacteria* UBA11222).

| **Gene** | **Feature** | **A_bin2** | **D_bin1** | **D_bin4** |
| --- | --- | --- | --- | --- |
| *badA* | Benzoate-CoA ligase |  | D_bin1_03340  D_bin1_04584  D_bin1_03340  D_bin1_04584 |  |
| *bcrC* | Benzoyl-CoA reductase subunit C |  | D_bin1_02559  D_bin1_04315  D_bin1_05164 |  |
| *bcrB* | Benzoyl-CoA reductase subunit B |  | D_bin1_02560  D_bin1_04316 |  |
| *bcrA* | Benzoyl-CoA reductase subunit A |  | D_bin1_02562  D_bin1_04318  D_bin1_04596 |  |
| *bcrD* | Benzoyl-CoA reductase subunit D |  | D_bin1_02561  D_bin1_04317 |  |
| *had* | 6-hydroxycyclohex-1-ene-1-carbonyl-CoA dehydrogenase |  | D_bin1_04587 |  |
| *oah* | 6-oxocyclohex-1-ene-carbonyl-CoA hydrolase |  | D_bin1_04586 |  |
| *badA* | Benzoate-CoA ligase |  | D_bin1_03340  D_bin1_04584  D_bin1_03340  D_bin1_04584 |  |
| *boxA* | Benzoyl-CoA 2,3-epoxidase subunit A |  | D_bin1_03346 |  |
| *boxB* | Benzoyl-CoA 2,3-epoxidase subunit B |  | D_bin1_03345 |  |
| *boxC* | Benzoyl-CoA-dihydrodiol lyase |  | D_bin1_03344 |  |
| *boxD* | 3,4-dehydroadipyl-CoA semialdehyde dehydrogenase |  | D_bin1_03342 |  |
| *benA* | Benzoate/toluate 1,2-dioxygenase subunit alpha | A_bin2_01595  A_bin2_01597  A_bin2_01853 |  | D_bin4_00253 |
| *benB* | Benzoate/toluate 1,2-dioxygenase subunit beta | A_bin2_01598  A_bin2_01854 |  | D_bin4_00252 |
| *benD* | Dihydroxycyclohexadiene carboxylate dehydrogenase | A_bin2_01591 |  |  |
| *dmpB* | Catechol 2,3-dioxygenase | A_bin2_01617  A_bin2_01842  A_bin2_01844  A_bin2_02065  A_bin2_02066 | D_bin1_00657 |  |
| *xylG* | 2-hydroxymuconate-6-semialdehyde dehydrogenase | A_bin2_00223  A_bin2_00616 | D_bin1_00664 | D_bin4_01568 |
| *xylH* | 4-oxalocrotonate tautomerase | A_bin2_00610 | D_bin1_00112 |  |
| *xylI* | 2-oxo-3-hexenedioate decarboxylase | A_bin2_00222  A_bin2_00611 | D_bin1_00666 | D_bin4_00638 |
| *xylF* | 2-hydroxymuconate-semialdehyde hydrolase | A_bin2_00365  A_bin2_02057 |  | D_bin4_00640 |
| *mhpD* | 2-keto-4-pentenoate hydratase |  |  | D_bin4_00639 |
| *xylJ* | 2-oxopent-4-enoate/cis-2-oxohex-4-enoate hydratase | A_bin2_00615 | D_bin1_00665  D_bin1_02643 |  |
| *mhpE* | 4-hydroxy 2-oxovalerate aldolase |  |  | D_bin4_00921 |
| *mhpF* | Acetaldehyde dehydrogenase |  |  | D_bin4_00922 |

Table S9. Genes encoding citrate cycle (TCA cycle) in A_bin2 (*Rugosibacter*), D_bin1 (*Azoarcus*), and D_bin4 (*Alphaproteobacteria* UBA11222).

| **Gene** | **Feature of gene product** | **A_bin2** | **D_bin1** | **D_bin4** |
| --- | --- | --- | --- | --- |
| *gltA* | Citrate synthase | A_bin2_01641  A_bin2_02044 | D_bin1_00215  D_bin1_04924 | D_bin4_01624 |
| *acnA* | Aconitate hydratase | A_bin2_01125 | D_bin1_00507  D_bin1_05342  D_bin1_05396 | D_bin4_00083 |
| *acnB* | Aconitate hydratase 2 / 2-methylisocitrate dehydratase | A_bin2_01650 | D_bin1_00506 |  |
| *IDH1* | Isocitrate dehydrogenase | A_bin2_01517 | D_bin1_01046  D_bin1_01047 | D_bin4_02245  D_bin4_02480 |
| *IDH3* | Isocitrate dehydrogenase (NAD+) | A_bin2_01965 |  | D_bin4_01942 |
| *OGDH* | 2-oxoglutarate dehydrogenase E1 component | A_bin2_01640 | D_bin1_00216  D_bin1_05136 | D_bin4_00272 |
| *DLST* | 2-oxoglutarate dehydrogenase E2 component | A_bin2_01639 | D_bin1_00217  D_bin1_05137 | D_bin4_00273 |
| *DLD* | Dihydrolipoamide dehydrogenase | A_bin2_01638  A_bin2_02347 | D_bin1_00218  D_bin1_00683  D_bin1_01547 | D_bin4_00021  D_bin4_00274 |
| *korA* | 2-oxoglutarate/2-oxoacid ferredoxin oxidoreductase subunit alpha |  | D_bin1_03029  D_bin1_03646  D_bin1_04058 | D_bin4_01641 |
| *korB* | 2-oxoglutarate/2-oxoacid ferredoxin oxidoreductase subunit beta |  | D_bin1_03028  D_bin1_03647  D_bin1_03848  D_bin1_04057  D_bin1_05218 | D_bin4_01642 |
| *sucD* | Succinyl-CoA synthetase alpha subunit | A_bin2_00740 | D_bin1_01326 | D_bin4_00271 |
| *sucC* | Succinyl-CoA synthetase beta subunit | A_bin2_00741 | D_bin1_01327 | D_bin4_00270  D_bin4_02657 |
| *sdhA* | Succinate dehydrogenase / fumarate reductase, flavoprotein subunit | A_bin2_01644 | D_bin1_00212 | D_bin4_01749 |
| *sdhB* | Succinate dehydrogenase / fumarate reductase, iron-sulfur subunit | A_bin2_01643 | D_bin1_00213  D_bin1_02726 | D_bin4_01748 |
| *sdhC* | Succinate dehydrogenase / fumarate reductase, cytochrome b subunit | A_bin2_01646 | D_bin1_00210  D_bin1_02728 | D_bin4_01751 |
| *sdhD* | Succinate dehydrogenase / fumarate reductase, membrane anchor subunit | A_bin2_01645 | D_bin1_00211  D_bin1_02727 | D_bin4_01750 |
| *frdA* | Fumarate reductase flavoprotein subunit |  | D_bin1_01363  D_bin1_04326 |  |
| *frdB* | Fumarate reductase iron-sulfur subunit |  | D_bin1_01364 |  |
| *frdC* | Fumarate reductase subunit C |  | D_bin1_01365 |  |
| *frdD* | Fumarate reductase subunit D |  | D_bin1_01366 |  |
| *fumAB* | Fumarate hydratase, class I | A_bin2_01862 | D_bin1_03506 |  |
| *fumC* | Fumarate hydratase, class II | A_bin2_01124 | D_bin1_00405 | D_bin4_00261 |
| *mdh* | Malate dehydrogenase | A_bin2_01648 | D_bin1_00208 | D_bin4_02656 |

Table S10. Genes encoding energy metabolism in A_bin2 (*Rugosibacter*), D_bin1 (*Azoarcus*), and D_bin4 (*Alphaproteobacteria* UBA11222).

| **Gene** | **Feature of gene product** | **A_bin2** | **D_bin1** | **D_bin4** |
| --- | --- | --- | --- | --- |
| *nuoA* | NADH-quinone oxidoreductase subunit A | A_bin2_00343 | D_bin1_00583 | |
| *nuoB* | NADH-quinone oxidoreductase subunit B | A_bin2_00344 | D_bin1_00582 | D_bin4_02985 |
| *nuoC* | NADH-quinone oxidoreductase subunit C | A_bin2_00345 | D_bin1_00581 | D_bin4_02986 |
| *nuoD* | NADH-quinone oxidoreductase subunit D | A_bin2_00346 | D_bin1_00580 | D_bin4_00502  D_bin4_02987 |
| *nuoE* | NADH-quinone oxidoreductase subunit E | A_bin2_00347 | D_bin1_00579 | D_bin4_00503  D_bin4_02988 |
| *nuoF* | NADH-quinone oxidoreductase subunit F | A_bin2_00348 | D_bin1_00578 | D_bin4_00504 |
| *nuoG* | NADH-quinone oxidoreductase subunit G | A_bin2_00349 | D_bin1_00577 | D_bin4_00505  D_bin4_00506 |
| *nuoH* | NADH-quinone oxidoreductase subunit H | A_bin2_00350 | D_bin1_00576 | D_bin4_00507 |
| *nuoI* | NADH-quinone oxidoreductase subunit I | A_bin2_00351 | D_bin1_00575 | D_bin4_00508 |
| *nuoJ* | NADH-quinone oxidoreductase subunit J | A_bin2_00352 | D_bin1_00574 | D_bin4_00509 |
| *nuoK* | NADH-quinone oxidoreductase subunit K | A_bin2_00353 | D_bin1_00573 | D_bin4_00510 |
| *nuoL* | NADH-quinone oxidoreductase subunit L | A_bin2_00354 | D_bin1_00572 | D_bin4_00511 |
| *nuoM* | NADH-quinone oxidoreductase subunit M | A_bin2_00355 | D_bin1_00571 | D_bin4_00512 |
| *nuoN* | NADH-quinone oxidoreductase subunit N | A_bin2_00356 | D_bin1_00570 | D_bin4_00513 |
| *cydA* | Cytochrome bd ubiquinol oxidase subunit I | A_bin2_00169 | | D_bin4_01361 |
| *cydB* | Cytochrome bd ubiquinol oxidase subunit II | A_bin2_00170 | | D_bin4_01362  D_bin4_02733 |
| *cydX* | Cytochrome bd-I ubiquinol oxidase subunit X | A_bin2_00171 | |  |
| *coxB* | Cytochrome c oxidase subunit II | A_bin2_00069 | D_bin1_01957  D_bin1_05280 | D_bin4_00119 |
| *coxA* | Cytochrome c oxidase subunit I | A_bin2_00068 | D_bin1_01958  D_bin1_05279 | D_bin4_00118 |
| *coxC* | Cytochrome c oxidase subunit III | A_bin2_00065 | D_bin1_01962  D_bin1_02455 | D_bin4_00114 |
| *ccoN* | Cytochrome c oxidase cbb3-type subunit I | A_bin2_01832 | D_bin1_02063 | |
| *ccoO* | Cytochrome c oxidase cbb3-type subunit II | A_bin2_01833 | D_bin1_02064 | |
| *ccoQ* | Cytochrome c oxidase cbb3-type subunit IV | A_bin2_01834 | D_bin1_02065 | |
| *ccoP* | Cytochrome c oxidase cbb3-type subunit III | A_bin2_01835 | D_bin1_02066  D_bin1_02229 | |
| *ATPF1A* | F-type H^+^/Na^+^-transporting ATPase subunit alpha | A_bin2_00521  A_bin2_00645 | D_bin1_02307 | D_bin4_02058 |
| *ATPF1B* | F-type H^+^/Na^+^-transporting ATPase subunit beta | A_bin2_00528  A_bin2_00643 | D_bin1_02309 | D_bin4_02056 |
| *ATPF1D* | F-type H^+^-transporting ATPase subunit delta | A_bin2_00646 | D_bin1_02306 | D_bin4_02059 |
| *ATPF1E* | F-type H^+^-transporting ATPase subunit epsilon | A_bin2_00527  A_bin2_00642 | D_bin1_02310 | D_bin4_02055 |
| *ATPF1G* | F-type H^+^-transporting ATPase subunit gamma | A_bin2_00520  A_bin2_00644 | D_bin1_02308 | D_bin4_02057 |
| *ATPF0A* | F-type H^+^-transporting ATPase subunit a | A_bin2_00525  A_bin2_00649  A_bin2_02480 | D_bin1_02303 | D_bin4_00577 |
| *ATPF0B* | F-type H^+^-transporting ATPase subunit b | A_bin2_00522  A_bin2_00647 | D_bin1_02305 | D_bin4_00579  D_bin4_00580 |
| *ATPF0C* | F-type H^+^-transporting ATPase subunit c | A_bin2_00524  A_bin2_00648 | D_bin1_02304 | D_bin4_00578 |


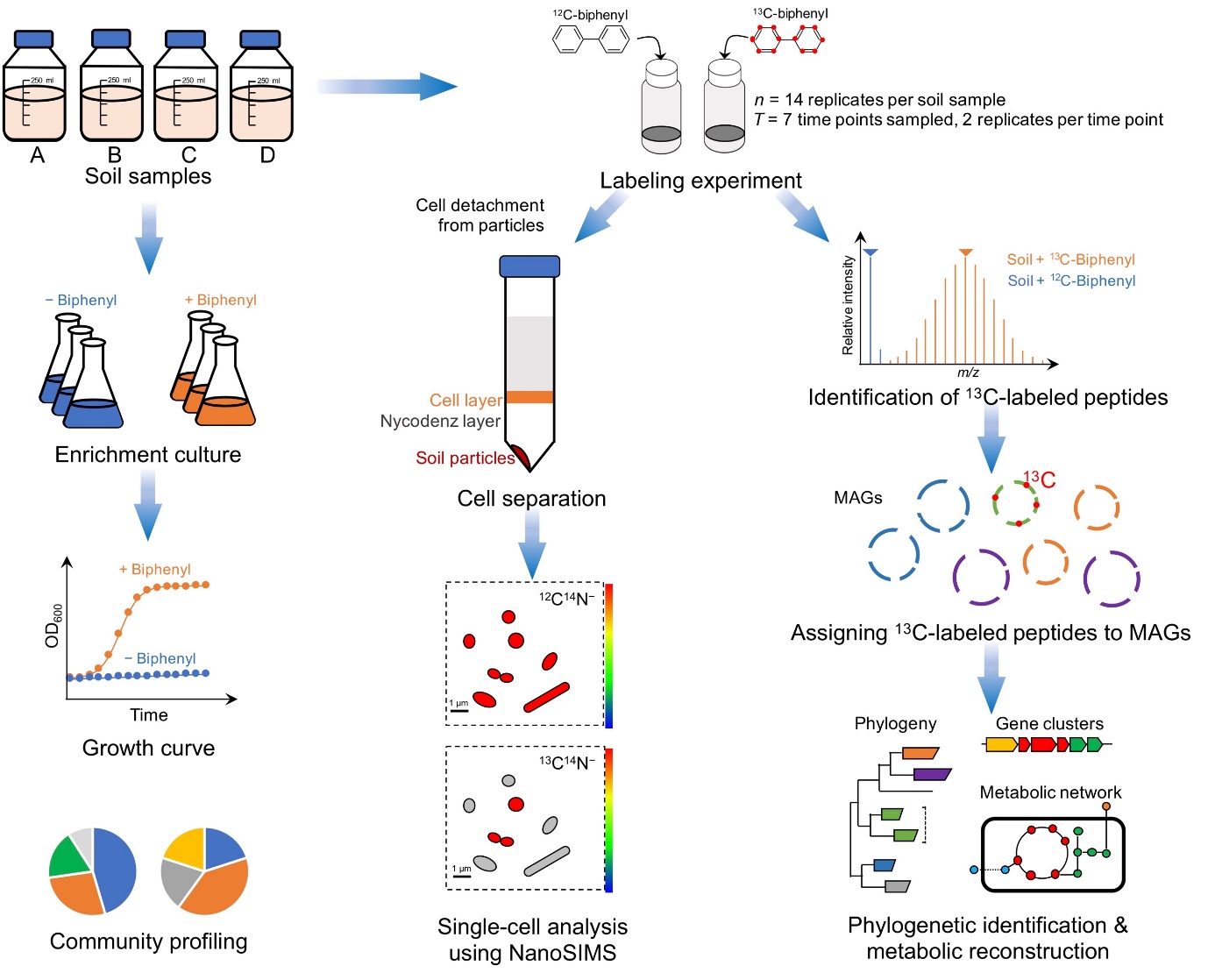


Figure S1. Experimental framework used to probe uncultured soil microorganisms responsible for aerobic biphenyl oxidation. Soil samples, collected via non-invasive procedures were assessed for the capacity of their microbial communities to oxidize biphenyl in a cultivation-dependent approach. The biphenyl-degrading enrichment cultures obtained were characterized by physiology experiments and 16S-rRNA-gene-based community profiling. Microcosm incubations of soils with ^13^C-labelled biphenyl in a short timeframe allowed the tracing of biphenyl-derived carbon into active microbial cells under near *in situ* conditions. Cells were detached from soil particles by treatment with tensioactive agents and shaking, and were separated from the soil matrix by density gradient centrifugation. Substrate uptake rates of indigenous microorganisms were resolved at single-cell level by nanoSIMS analyses. The full genomic potential of biphenyl degraders was resolved by isotope-specific metaproteomics and metagenomics. ^13^C-labelled peptides were identified by comparing the mass spectra of peptides from labelled and unlabeled microcosms; they were further assigned to metagenome assembled genomes (MAGs) recovered from the corresponding soils. Genomic analyses of ^13^C-labelled MAGs revealed the phylogenetic identity and metabolic pathways of microorganisms catalyzing biphenyl oxidation in soil environments.


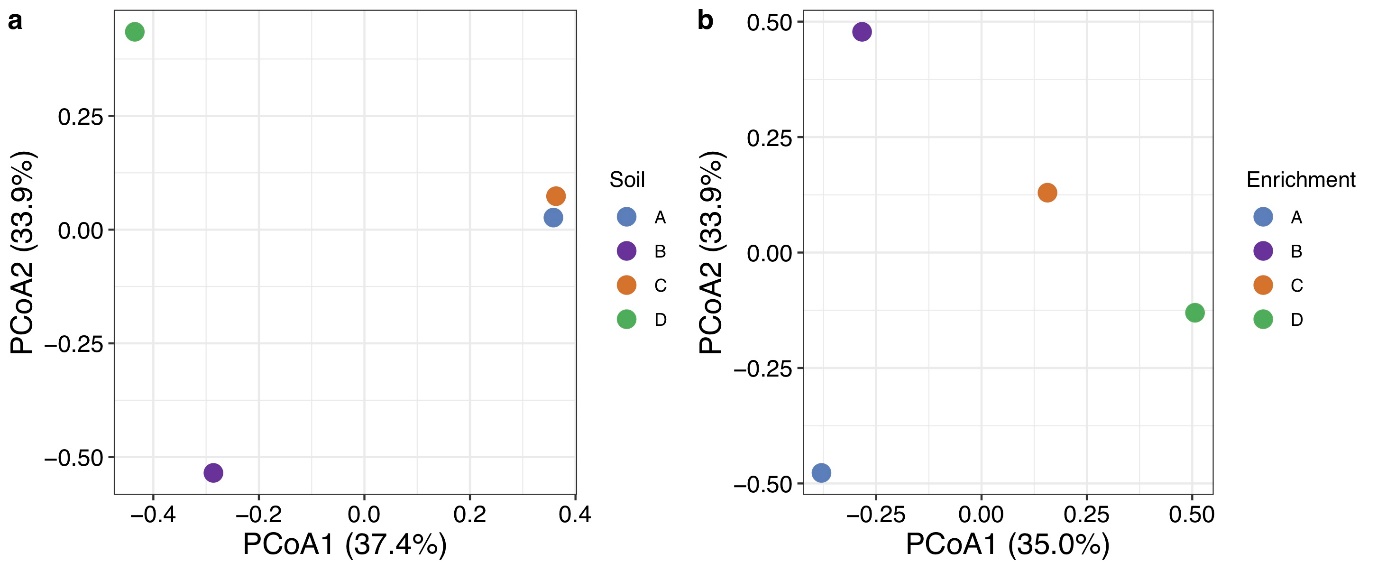


Figure S2. Comparison of microbial community structure of the soils (a) and corresponding enrichment cultures (b). Principal coordinates analysis (PCoA) suggests that the two river sediments (A and C) shared more similar microbial communities among each other than to the other soils (B and D). No obvious pattern of microbial community composition was observed for the four biphenyl-degrading enrichment cultures. PCoA was performed on Bray-Curtis dissimilarity between samples. The percentage of variation explained by the plotted principal coordinates is indicated on the axes.


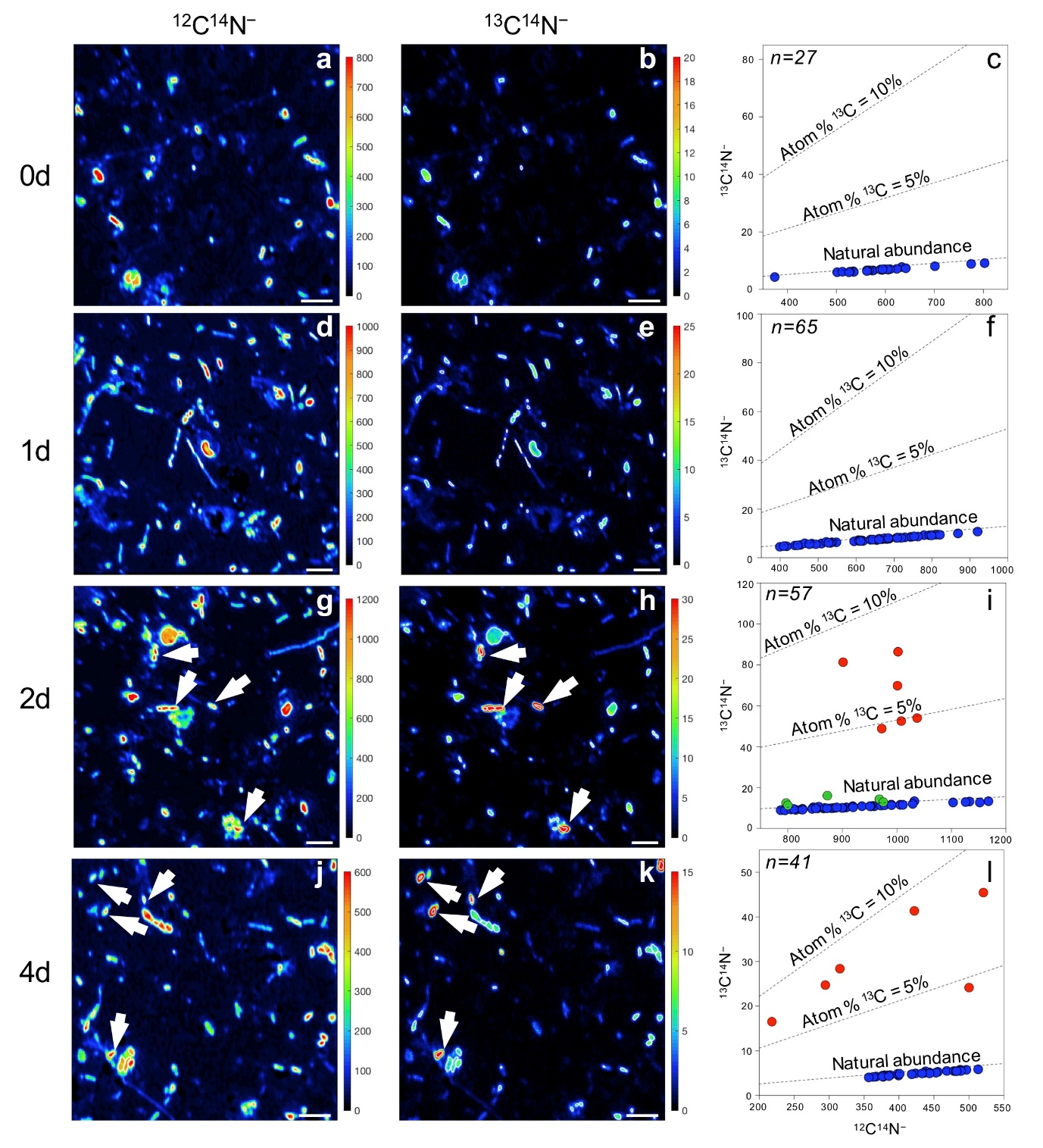


Figure S3. NanoSIMS analysis of cells extracted from soil A after incubation with ^13^C-labelled biphenyl for 0 (a-c), 1 (d-f), 2 (g-i), and 4 days (j-l). Secondary ion images of ^12^C^14^N^-^ (a, d, g, and j) and ^13^C^14^N^-^ (b, e, h, k) were used to identify individual cells and to quantify ^13^C abundance. Arrows in ^12^C^14^N^-^ ion images (g and j) point to cells enriched in ^13^C in panel i and l; Raw ion counts of ^12^C^14^N^-^ and ^13^C^14^N^-^ for individual cells were shown as scatter plot (c, f, i, and l). Cells having ^13^C abundance over 2% are represented as red symbols (f), whereas all other cells are represented as blue symbols (c, f). Scale bars = 3.5 μm.


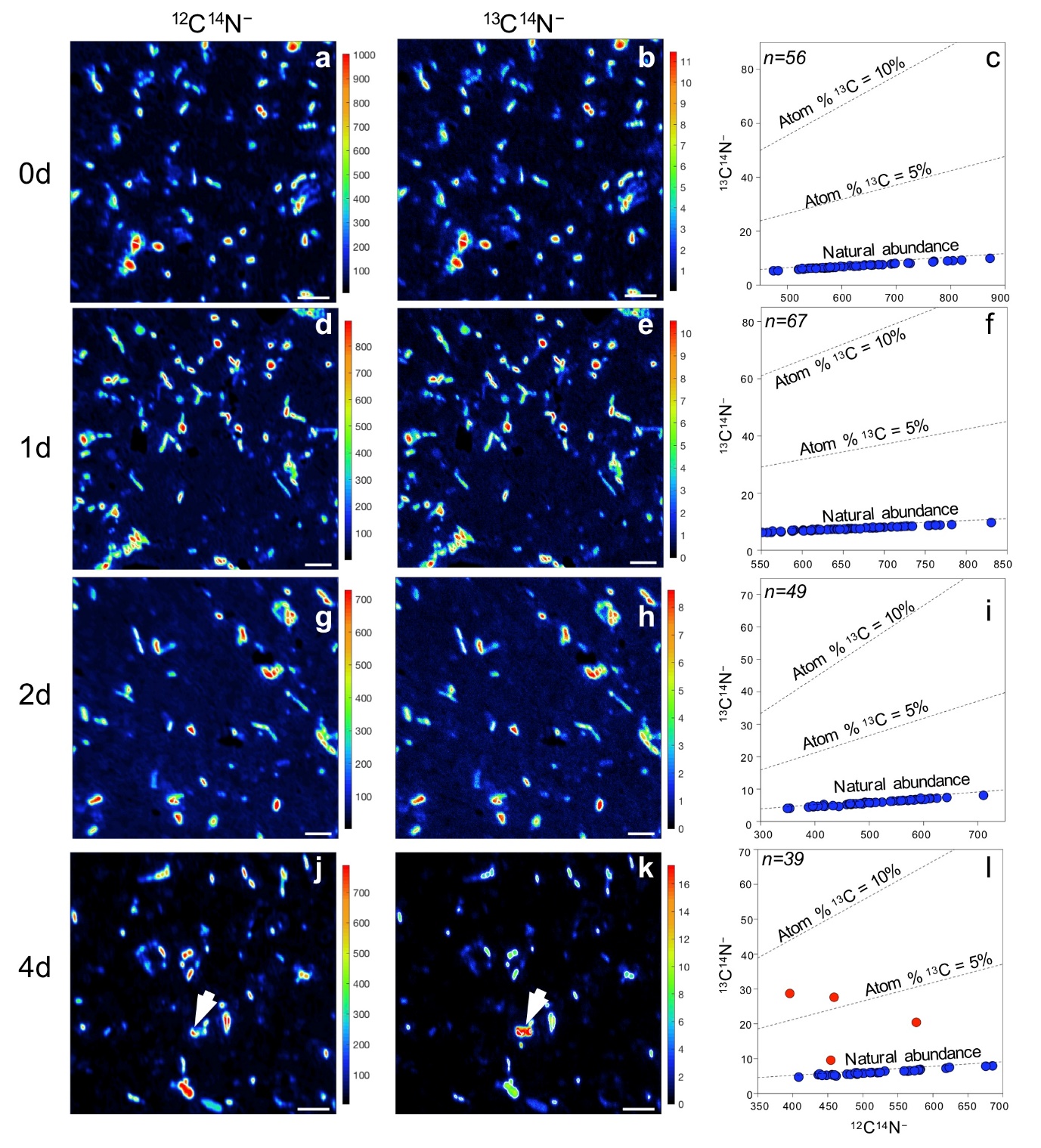


Figure S4. NanoSIMS analysis of cells extracted from soil B after incubation with ^13^C-labelled biphenyl for 0 (a-c), 1 (d-f), 2 (g-i), and 4 days (j-l). Secondary ion images of ^12^C^14^N^-^ (a, d, g, and j) and ^13^C^14^N^-^ (b, e, h, k) were used to identify individual cells and to quantify ^13^C abundance. Arrows in ^12^C^14^N^-^ ion images (g and j) point to cells enriched in ^13^C in panel i and l; Raw ion counts of ^12^C^14^N^-^ and ^13^C^14^N^-^ for individual cells were shown as scatter plot (c, f, i, and l). Cells having ^13^C abundance over 2% are represented as red symbols (f), whereas all other cells are represented as blue symbols (c, f). Scale bars = 3.5 μm.


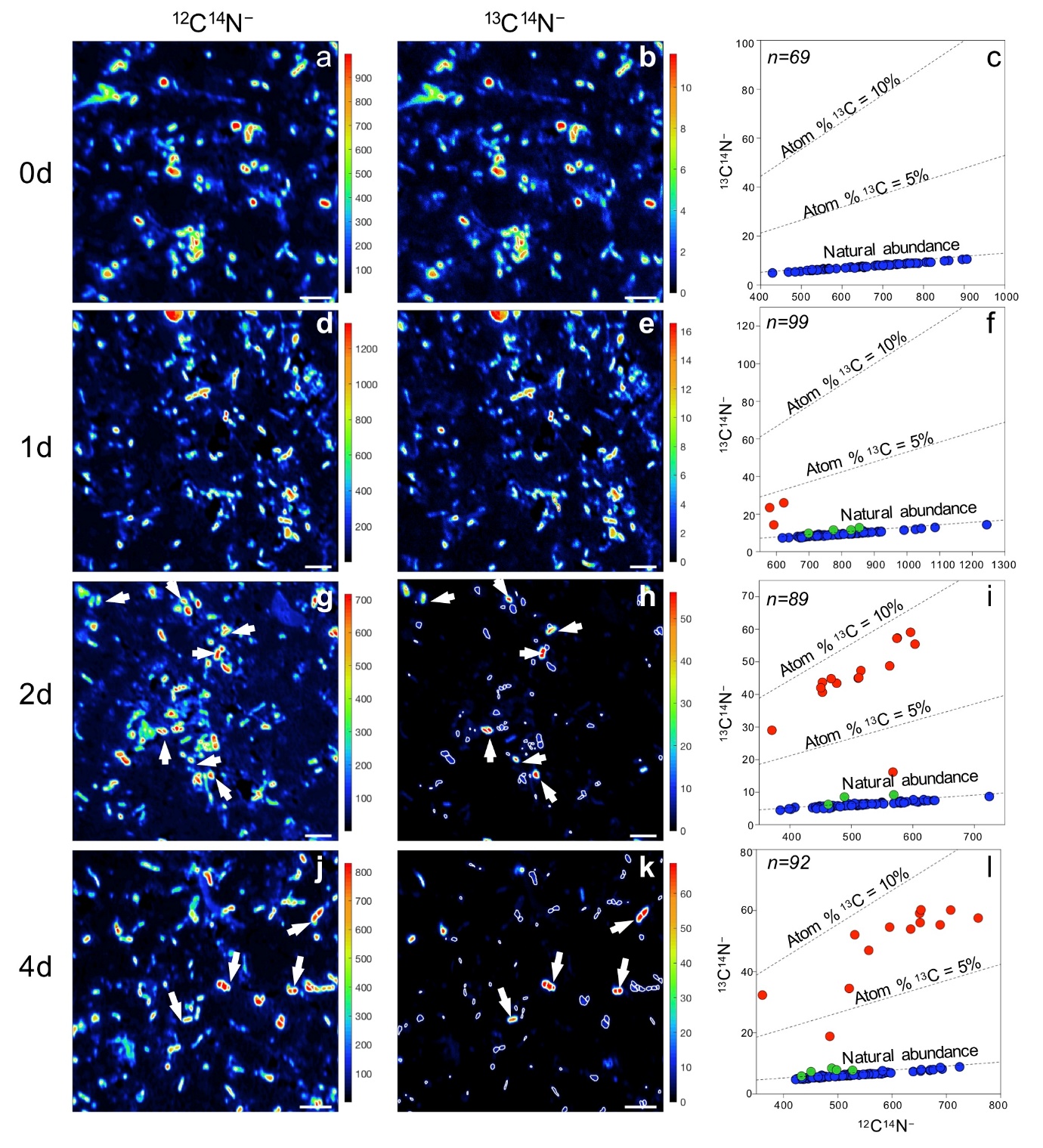


Figure S5. NanoSIMS analysis of cells extracted from soil C after incubation with ^13^C-labelled biphenyl for 0 (a-c), 1 (d-f), 2 (g-i), and 4 days (j-l). Secondary ion images of ^12^C^14^N^-^ (a, d, g, and j) and ^13^C^14^N^-^ (b, e, h, k) were used to identify individual cells and to quantify ^13^C abundance. Arrows in ^12^C^14^N^-^ ion images (g and j) point to cells enriched in ^13^C in panel i and l; Raw ion counts of ^12^C^14^N^-^ and ^13^C^14^N^-^ for individual cells were shown as scatter plot (c, f, i, and l). Cells having ^13^C abundance over 2% are represented as red symbols (f), whereas all other cells are represented as blue symbols (c, f). Scale bars = 3.5 μm.


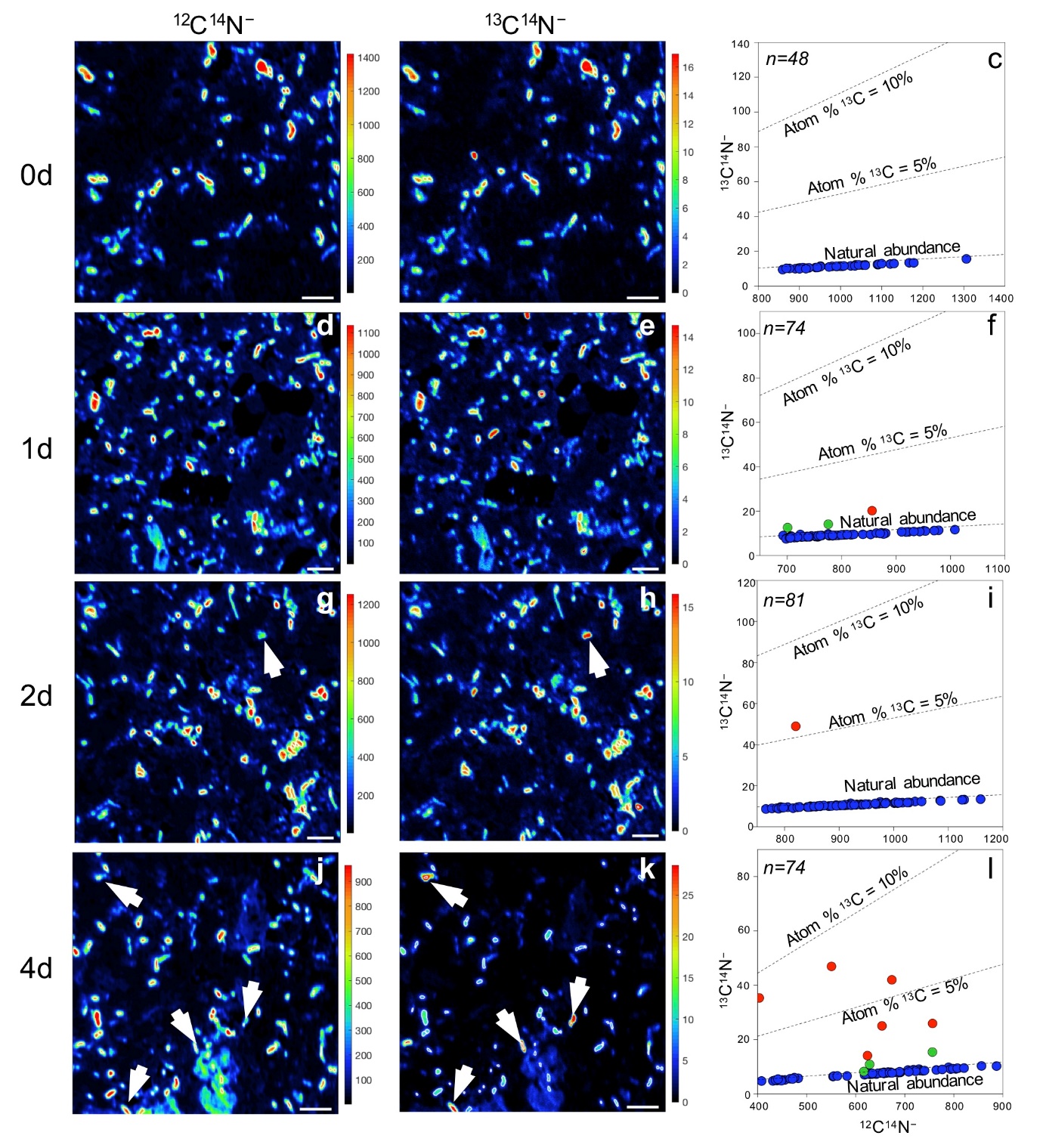


Figure S6. NanoSIMS analysis of cells extracted from soil D after incubation with ^13^C-labelled biphenyl for 0 (a-c), 1 (d-f), 2 (g-i), and 4 days (j-l). Secondary ion images of ^12^C^14^N^-^ (a, d, g, and j) and ^13^C^14^N^-^ (b, e, h, k) were used to identify individual cells and to quantify ^13^C abundance. Arrows in ^12^C^14^N^-^ ion images (g and j) point to cells enriched in ^13^C in panel i and l; Raw ion counts of ^12^C^14^N^-^ and ^13^C^14^N^-^ for individual cells were shown as scatter plot (c, f, i, and l). Cells having ^13^C abundance over 2% are represented as red symbols (f), whereas all other cells are represented as blue symbols (c, f). Scale bars = 3.5 μm.


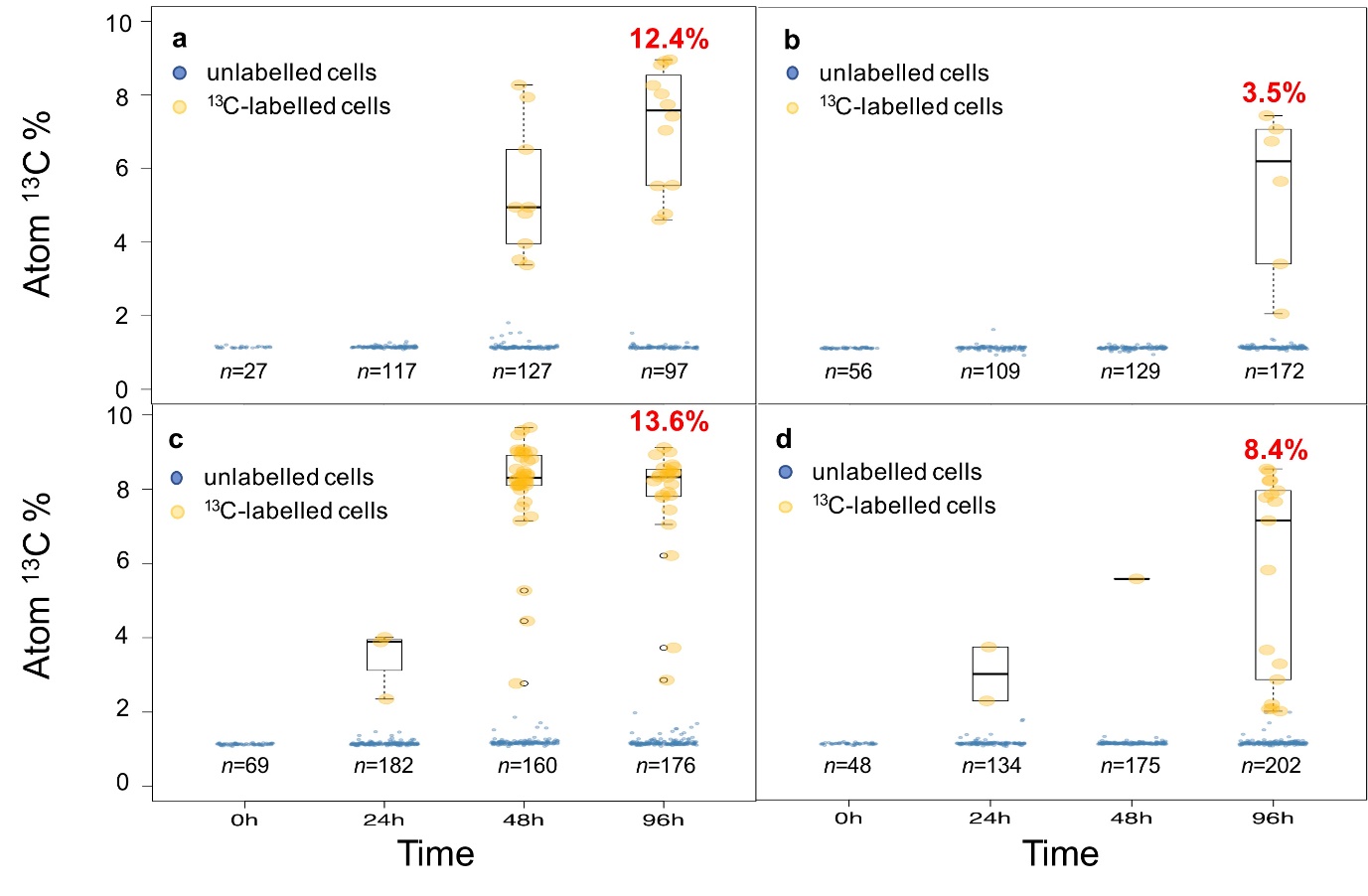


Figure S7. ^13^C abundance of cells extracted from the four soils (A, B, C, and D) after incubation with ^13^C-labelled biphenyl for 0, 24, 48, and 96 hours. Each dot represents ^13^C abundance of a single cell calculated from raw ion counts of ^12^C^14^N^−^ and ^13^C^14^N^−^. The number of cells analyzed at each time point was indicated below the dots. Cells having ^13^C abundance over 2% are represented as yellow symbols, whereas all other cells are represented as blue symbols. Boxplots summarize the statistical distribution of ^13^C abundance (range, median, 25%, and 75% quantiles) for all labelled cells at a given time point in each soil. The relative abundance of ^13^C-labeleld cells after 96 hours’ incubation are indicated above the box. The data was compiled from 1 field of view of nanoSIMS analysis (FoV) for 0 hour, 2 FoVs for 24 and 48 hours, and 3 FoVs for 96 hours.


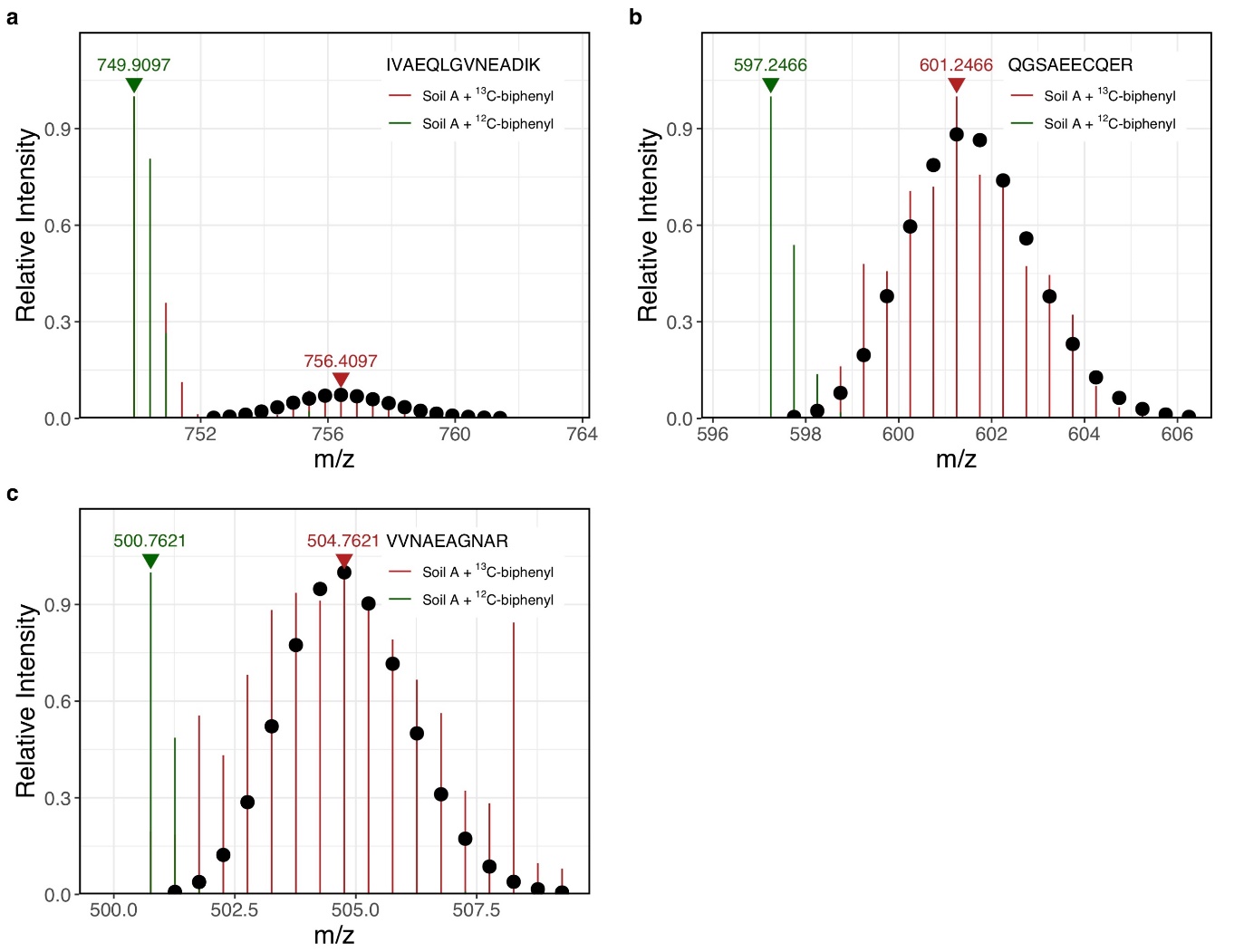


Figure S8. ^13^C-peptides that were mapped unambiguously to A_bin2 (*Rugosibacter*). Mass spectra of the peptide retrieved from ^13^C- and ^12^C-biphenyl incubated microcosms were shown as red and green lines. The most abundant isotopomer for both microcosms was indicated by arrow and peak *m/z*. Empty circles represented the best fit of the theoretical isotopic patterns. ^13^C incorporation levels for peptides IVAEQLGVNEADIK, QGSAEECQER, and VVNAEAGNAR were estimated to be 19.8%, 19%, and 18.4%, respectively.


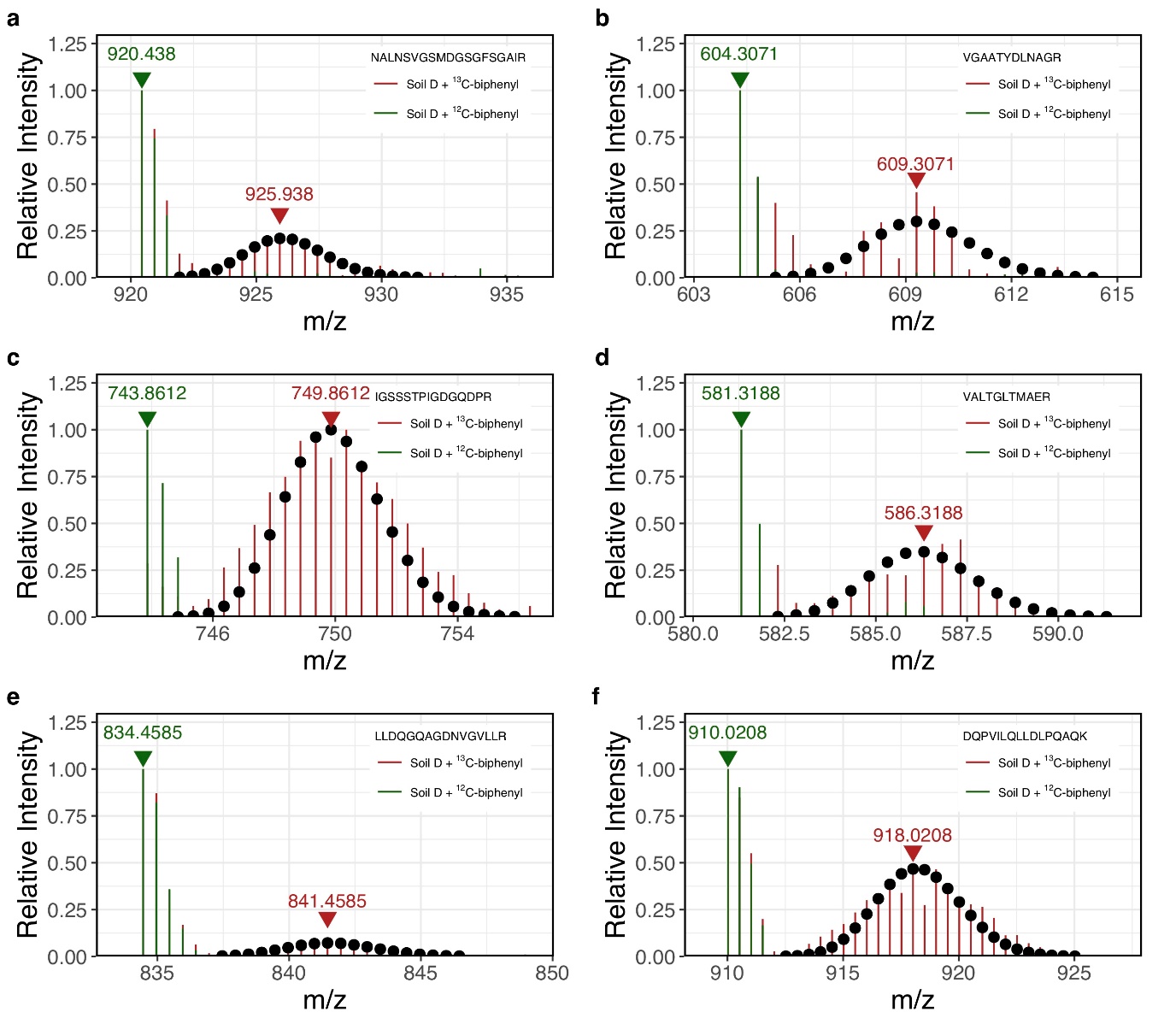


Figure S9. ^13^C-peptides that were mapped unambiguously to D_bin1 (*Azoarcus*). Mass spectra of the peptide retrieved from ^13^C- and ^12^C-biphenyl incubated microcosms were shown as red and green lines. The most abundant isotopomer for both microcosms was indicated by arrow and peak *m/z*. Empty circles represented the best fit of the theoretical isotopic patterns. ^13^C incorporation levels for peptides NALNSVGSMDGSGFSGAIR, VGAATYDLNAGR, IGSSSTPIGDGQDPR, VALTGLTMAER, LLDQGQAGDNVGVLLR, and DQPVILQLLDLPQAQK were estimated to be 14.7%, 19.5%, 19.8%, 21.2%, 19.2%, and 20.2%, respectively.


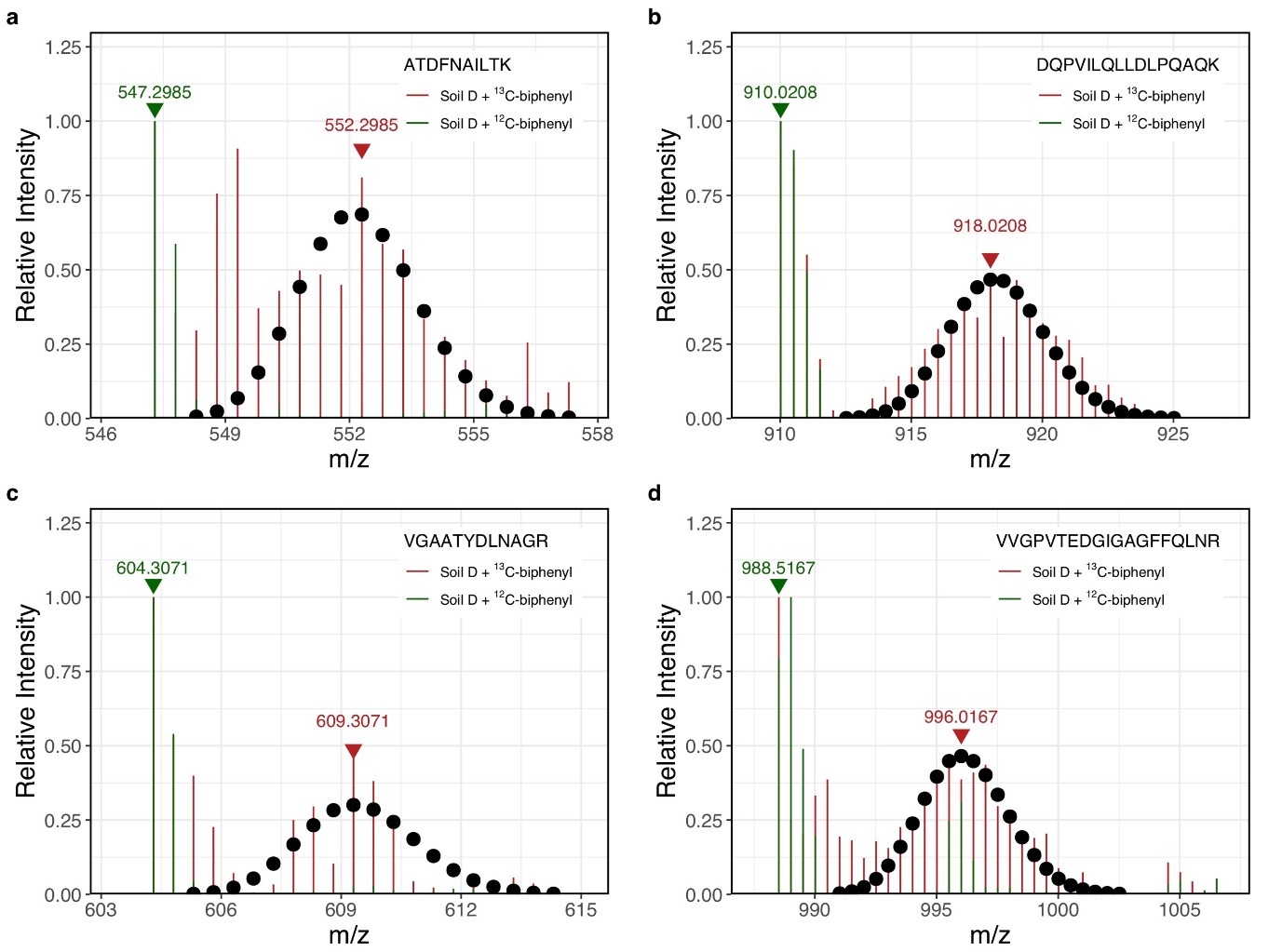


Figure S10. ^13^C-peptides that were mapped unambiguously to D_bin4 (*Alphaproteobacteria UBA11222*). Mass spectra of the peptide retrieved from ^13^C- and ^12^C-biphenyl incubated microcosms were shown as red and green lines. The most abundant isotopomer for both microcosms was indicated by arrow and peak *m/z*. Empty circles represented the best fit of the theoretical isotopic patterns. ^13^C incorporation levels for peptides ATDFNAILTK, DQPVILQLLDLPQAQK, VGAATYDLNAGR, and VVGPVTEDGIGAGFFQLNR were estimated to be 21%, 20.2%, 19.5%, and 17%, respectively.


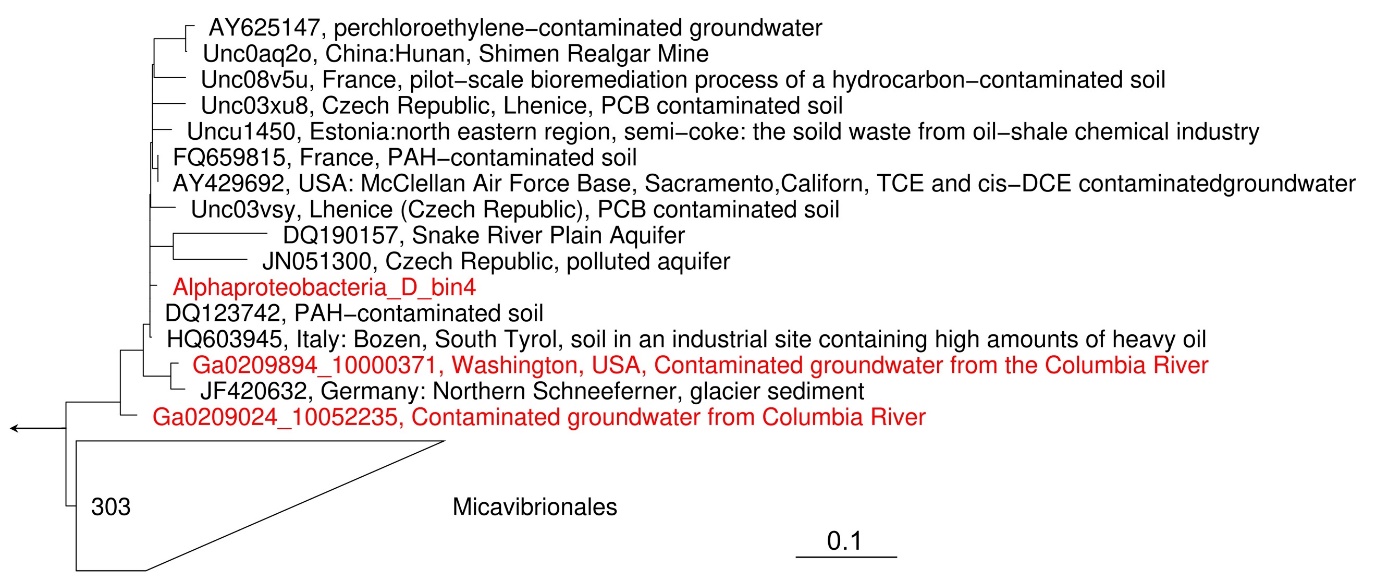


Figure S11. Consensus tree of Alphaproteobacteria_D_bin4 phylotypes and its biogeographic distribution. Phylogenetic tree was reconstructed using SILVA database (v132; Ref NR99). Partial 16S rRNA gene sequences from three metagenome-assembled genomes (shown in red) were placed to the reference tree of life using ARB software (‘Add Species to Existing Tree’ function).


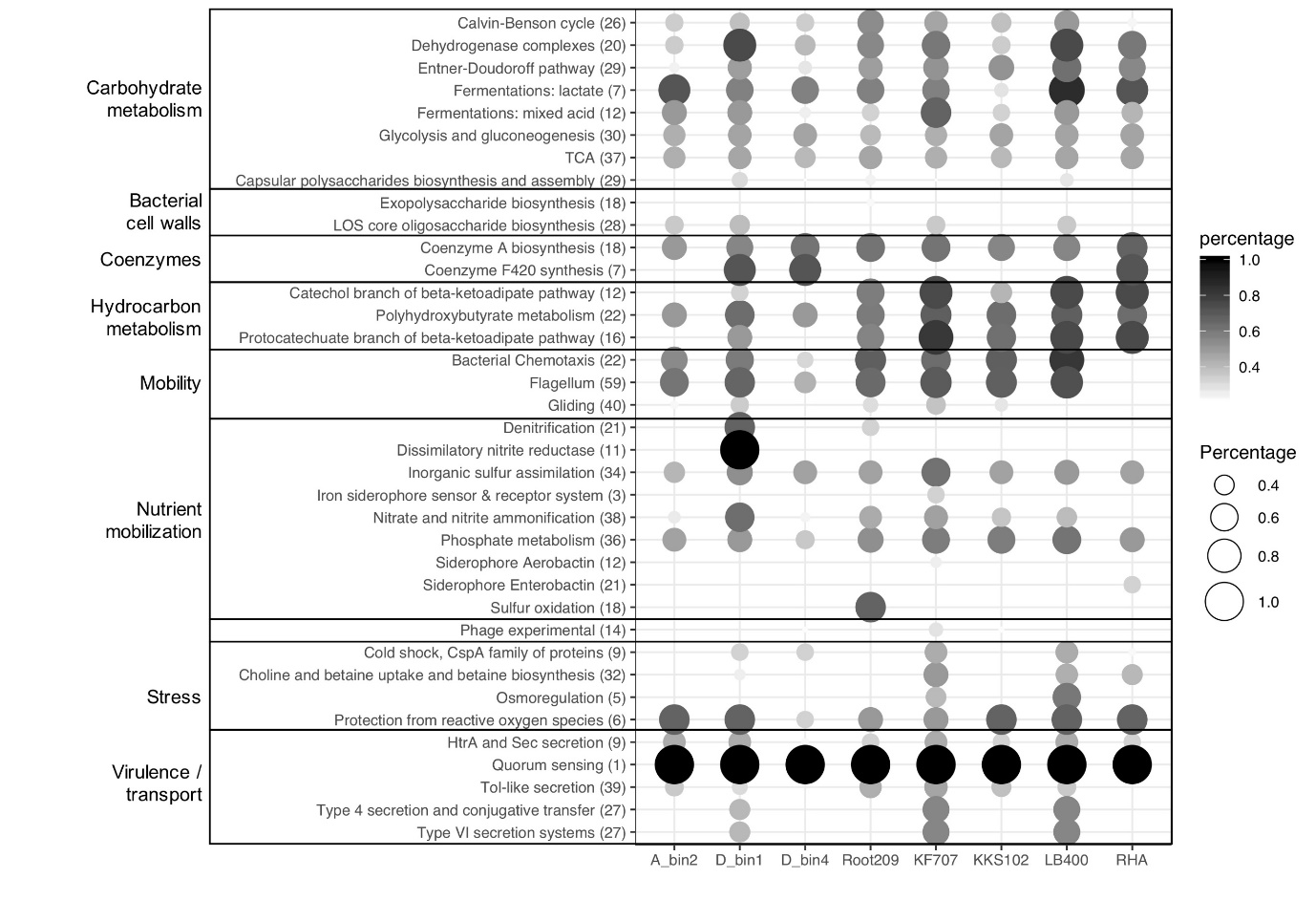


Figure S12. Abundance of functional categories detected in the MAGs recovered from soils (A_bin2, D_bin1, and D_bin4) and in cultivated biphenyl-degraders (Root209, KF707, KKS102, LB400, and RHA). Functional categories are based on the SEED subsystems and were compared against genes annotated within each selected genome using blastp (e-value cutoff of 1 × 10^−30^; query coverage cutoff of 70%; identity cutoff of 30%). Numbers in parentheses: total number of genes within each functional category. Percentage: percent of total number of genes per subsystem found within each genome. A_bin2: Rugosibacter_A_bin2; D_bin1: Azoarcus_D_bin1; D_bin4: Alphaproteobacteria_D_bin4; Root209: *Hydrogenophage* sp. Root209; KF707: *Pseudomonas furukawaii* KF707; KKS102: *Acidovorax* sp. KKS102; LB400: *Burkholderia xenovorans* LB400; RHA: *Rhodococcus jostii* RHA1.

**References**

1. Fukuda M, Yasukochi Y, Kikuchi Y, Nagata Y, Kimbara K, Horiuchi H, et al. Identification of the bphA and bphB genes of *Pseudomonas* sp. Strain KKS102 involved in degradation of biphenyl and polychlorinated biphenyls. Biochem Biophys Res Commun. 1994;202(2):850-6.

2. Meynet P, Head IM, Werner D, Davenport RJ. Re-evaluation of dioxygenase gene phylogeny for the development and validation of a quantitative assay for environmental aromatic hydrocarbon degraders. FEMS Microbiol Ecol. 2015;91(6).

3. Mukerjee-Dhar G, Shimura M, Miyazawa D, Kimbara K, Hatta T. Bph genes of the thermophilic PCB degrader, *Bacillus* sp. JF8: characterization of the divergent ring-hydroxylating dioxygenase and hydrolase genes upstream of the Mn-dependent BphC. Microbiology. 2005;151(12):4139-51.

4. Erickson BD, Mondello FJ. Nucleotide sequencing and transcriptional mapping of the genes encoding biphenyl dioxygenase, a multicomponent polychlorinated-biphenyl-degrading enzyme in *Pseudomonas* strain LB400. J Bacteriol. 1992;174(9):2903-12.

5. Hiraoka Y, Yamada T, Tone K, Futaesaku Y, Kimbara K. Flow cytometry analysis of changes in the DNA content of the polychlorinated biphenyl degrader *Comamonas testosteroni* TK102: effect of metabolites on cell-cell separation. Appl Environ Microbiol. 2002;68(10):5104-12.

6. Watanabe T, Yamazoe A, Hosoyama A, Fujihara H, Suenaga H, Hirose J, et al. Draft genome sequence of *Cupriavidus pauculus* Strain KF709, a biphenyl-utilizing bacterium isolated from biphenyl-contaminated soil. Genome Announc. 2015;3(2).

7. Ilori MO, Picardal FW, Aramayo R, Adebusoye SA, Obayori OS, Benedik MJ. Catabolic plasmid specifying polychlorinated biphenyl degradation in *Cupriavidus* sp. strain SK-4: mobilization and expression in a pseudomonad. J Basic Microbiol. 2015;55(3):338-45.

8. Nguyen PA, Trinh TH, Fukumitsu Y, Shimodaira J, Miyauchi K, Tokuda M, et al. Gene cluster and regulation system for 1,1-dichloro-2,2-bis(4-chlorophenyl)ethylene (DDE) degradation in *Janibacter* sp. TYM3221. J Biosci Bioeng. 2013;116(1):91-100.

9. Sylvestre M, Sirois M, Hurtubise Y, Bergeron J, Ahmad D, Shareck F, et al. Sequencing of *Comamonas testosteroni* strain B-356 biphenyl/chlorobiphenyl dioxygenase genes: evolutionary relationships among Gram-negative bacterial biphenyl dioxygenases. Gene. 1996;174(2):195-202.

10. Kimura N, Watanabe T, Suenaga H, Fujihara H, Futagami T, Goto M, et al. *Pseudomonas furukawaii* sp. nov., a polychlorinated biphenyl-degrading bacterium isolated from biphenyl-contaminated soil in Japan. Int J Syst Evol Microbiol. 2018;68(5):1429-35.

11. Taira K, Hirose J, Hayashida S, Furukawa K. Analysis of bph operon from the polychlorinated biphenyl-degrading strain of *Pseudomonas pseudoalcaligenes* KF707. J Biol Chem. 1992;267(7):4844-53.

12. Li Q, Wang X, Yin G, Gai Z, Tang H, Ma C, et al. New metabolites in dibenzofuran cometabolic degradation by a biphenyl-cultivated *Pseudomonas putida* strain B6-2. Environ Sci Technol. 2009;43(22):8635-42.

13. Haddock JD, Gibson DT. Purification and characterization of the oxygenase component of biphenyl 2,3-dioxygenase from *Pseudomonas* sp. strain LB400. J Bacteriol. 1995;177(20):5834-9.

14. Master ER, Mohn WW. Induction of bphA, encoding biphenyl dioxygenase, in two polychlorinated biphenyl-degrading bacteria, psychrotolerant *Pseudomonas* strain Cam-1 and mesophilic *Burkholderia* strain LB400. Appl Environ Microbiol. 2001;67(6):2669-76.

15. Toussaint A, Merlin C, Monchy S, Benotmane MA, Leplae R, Mergeay M, et al. The biphenyl- and 4-chlorobiphenyl-catabolic transposon Tn4371, a member of a new family of genomic islands related to IncP and Ti plasmids. Appl Environ Microbiol. 2003;69(8):4837-45.

16. Taguchi K, Motoyama M, Iida T, Kudo T. Polychlorinated biphenyl/biphenyl degrading gene clusters in *Rhodococcus* sp. K37, HA99, and TA431 are different from well-known bph gene clusters of Rhodococci. Biosci Biotechnol Biochem. 2007;71(5):1136-44.

17. Kosono S, Maeda M, Fuji F, Arai H, Kudo T. Three of the seven bphC genes of *Rhodococcus erythropolis* TA421, isolated from a termite ecosystem, are located on an indigenous plasmid associated with biphenyl degradation. Applied Environ Microbiol. 1997;63(8):3282.

18. Asturias JA, Díaz E, Timmis KN. The evolutionary relationship of biphenyl dioxygenase from Gram-positive *Rhodococcus globerulus* P6 to multicomponent dioxygenases from Gram-negative bacteria. Gene. 1995;156(1):11-8.

19. Yang X, Liu X, Song L, Xie F, Zhang G, Qian S. Characterization and functional analysis of a novel gene cluster involved in biphenyl degradation in *Rhodococcus* sp. strain R04. J Appl Microbiol. 2007;103(6):2214-24.

20. Masai E, Yamada A, Healy JM, Hatta T, Kimbara K, Fukuda M, et al. Characterization of biphenyl catabolic genes of Gram-positive polychlorinated biphenyl degrader *Rhodococcus* sp. strain RHA1. Appl Environ Microbiol. 1995;61(6):2079.

21. Chadhain SM, Moritz EM, Kim E, Zylstra GJ. Identification, cloning, and characterization of a multicomponent biphenyl dioxygenase from *Sphingobium yanoikuyae* B1. J Ind Microbiol Biotechnol. 2007;34(9):605-13.

22. Romine MF, Stillwell LC, Wong K-K, Thurston SJ, Sisk EC, Sensen C, et al. Complete sequence of a 184-Kilobase catabolic plasmid from *Sphingomonas aromaticivorans* F199. J Bacteriol. 1999;181(5):1585.
